# Local delivery of cell surface-targeted immunocytokines programs systemic antitumor immunity

**DOI:** 10.1038/s41590-024-01925-7

**Published:** 2024-08-07

**Authors:** Luciano Santollani, Laura Maiorino, Yiming J. Zhang, Joseph R. Palmeri, Jordan A. Stinson, Lauren R. Duhamel, Kashif Qureshi, Jack R. Suggs, Owen T. Porth, William Pinney, Riyam Al Msari, Agnes A. Walsh, K. Dane Wittrup, Darrell J. Irvine

**Affiliations:** 1 Department of Chemical Engineering, Massachusetts Institute of Technology, Cambridge, MA USA; 2 Koch Institute for Integrative Cancer Research, Massachusetts Institute of Technology, Cambridge, MA USA; 3 Howard Hughes Medical Institute, Chevy Chase, MD USA; 4 Department of Biological Engineering, Massachusetts Institute of Technology, Cambridge, MA USA; 5 Ragon Institute of Massachusetts General Hospital, Massachusetts Institute of Technology, and Harvard University, Cambridge, MA USA; 6 Department of Materials Science and Engineering, Massachusetts Institute of Technology, Cambridge, MA USA

**Keywords:** Cancer immunotherapy, Translational immunology, Tumour immunology

## Abstract

Systemically administered cytokines are potent immunotherapeutics but can cause severe dose-limiting toxicities. To overcome this challenge, cytokines have been engineered for intratumoral retention after local delivery. However, despite inducing regression of treated lesions, tumor-localized cytokines often elicit only modest responses at distal untreated tumors. In the present study, we report a localized cytokine therapy that safely elicits systemic antitumor immunity by targeting the ubiquitous leukocyte receptor CD45. CD45-targeted immunocytokines have lower internalization rates relative to wild-type counterparts, leading to sustained downstream *cis* and *trans* signaling between lymphocytes. A single intratumoral dose of αCD45-interleukin (IL)-12 followed by a single dose of αCD45-IL-15 eradicated treated tumors and untreated distal lesions in multiple syngeneic mouse tumor models without toxicity. Mechanistically, CD45-targeted cytokines reprogrammed tumor-specific CD8^+^ T cells in the tumor-draining lymph nodes to have an antiviral transcriptional signature. CD45 anchoring represents a broad platform for protein retention by host immune cells for use in immunotherapy.

## Abstract (web summary)

Cytokine therapies have been used to treat cancer but are susceptible to adverse effects owing to the activation of systemic inflammatory pathways. Here the authors avoid this toxicity by developing CD45-targeted IL-12 and IL-15 immunocytokines that are effective in treating tumors in mice when dosed intratumorally.

## Main

Cytokines are a class of small proteins that serve as critical modulators of immune signaling cascades. As a result of their multifunctional roles in lymphocyte biology, cytokines have long been recognized as promising cancer immunotherapy agents^1–3^. IL-2 was the first efficacious immunotherapy for advanced cancer, eliciting a 17% overall response rate as a monotherapy in metastatic melanoma^4^. Despite this clinical validation, dose-limiting toxicities greatly hinder the clinical utilization of cytokines for oncology. This can be attributed to systemic lymphocyte activation triggered by the high doses that must be administered to achieve meaningful tumor concentrations.

To overcome this challenge, protein engineering has emerged as a tool to address the shortcomings of cytokine therapies^5–7^. One strategy to increase both safety and efficacy is to engineer cytokines for local delivery and retention within tumors and/or tumor-draining lymph nodes (TDLNs). We and others have previously demonstrated that potent cytokines and other immune agonists that are highly toxic when administered systemically can safely and effectively drive antitumor immunity when administered intratumorally (i.t.) to accessible lesions, by ‘anchoring’ these drugs to either intratumoral collagen or injected materials such as liposomes or alum particles^8–14^. These approaches are enabled by modern interventional radiology methods that make intratumoral administration feasible for most cancers^14–16^. In both preclinical models and early stage clinical trials, intratumoral therapies have elicited profound regressions of injected lesions, but they typically elicit modest responses at distal untreated sites^15,17^. Collectively, these data raise the question of whether localized immunotherapies are capable of eliciting robust systemic responses against metastatic disease.

In the present study, we report a localized immunotherapy regimen targeting cytokines to the universal leukocyte receptor CD45. We apply this cell-surface targeting to two well-studied cytokines, IL-12 and IL-15. CD45-targeted immunocytokines are retained on the cell surface and trigger enhanced and prolonged signaling relative to their native cytokine counterparts. Optimizing dose level, tissue localization and timing results in a highly efficacious and nontoxic cytokine therapy capable of eradicating treated tumors, as well as eliciting complete responses at distal lesions in multiple syngeneic models. A single dose of CD45-targeted IL-12 concentrated in the tumor, followed by a single dose of CD45-targeted IL-15 accumulated in both the tumor and the TDLNs, altered T cell programming in the TDLNs, expanding tumor-specific CD8^+^ T cells with a transcriptional signature mirroring effective responses to acute viral infection. Dissemination of this potent effector T cell pool led to systemic antitumor immunity characterized by complete responses in both treated and untreated tumors.

## Results

### Engineering CD45 immunocytokines

**Fig. 1:**
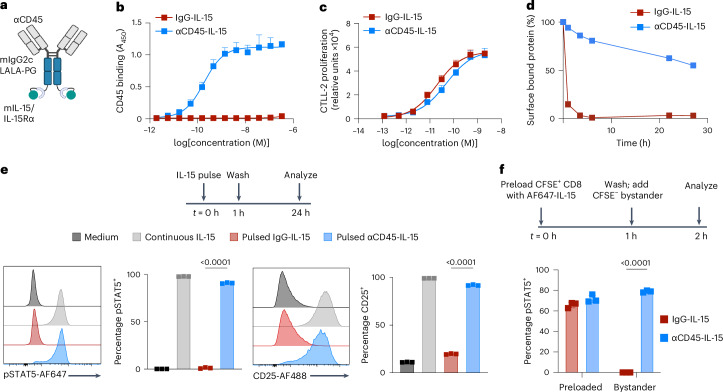
CD45 immunocytokines are retained on the cell surface and display extended signaling. **a**, Schematic of IL-15 immunocytokine (created using BioRender.com). **b**, ELISA absorbance measurement (*n* = 2 biological replicates) of αCD45-IL-15 and IgG-IL-15 binding to plate-bound mouse CD45. **c**, Luminescence measurement of CTLL-2 cell proliferation (*n* = 3 biological replicates) after 48-h incubation with IgG-IL-15 or αCD45-IL-15 at the indicated concentrations. **d**, Internalization kinetics of IgG-IL-15 or αCD45-IL-15 labeled with AF488 after binding to primary activated CD8^+^ T cells (*n* = 3 biological replicates). Surface signal was calculated by fluorescence quenching with an αAF488 antibody. **e**, Primary CD8 T cells (*n* = 3 biological replicates) were pulsed for 1 h with IL-15 immunocytokine fusions. Cells were washed to remove unbound cytokine and, 24 h later, fixed, permeabilized and stained for flow cytometry analysis of pSTAT5 and CD25. **f**, Primary CD8^+^ T cells (*n* = 3 biological replicates) labeled with CFSE and incubated with AF647-labeled IL-15 immunocytokine fusions for 1 h (‘preloaded’), washed and mixed into nonlabeled ‘bystander’ cells for 1 h at a 1:20 preloaded:bystander ratio. Cells were then fixed, permeabilized and stained for flow cytometry analysis of pSTAT5. Bystander cells were defined as CFSE^−^AF647^−^. In **b**–**f**, data are mean ± s.d. from stated replicates. *P* values were determined by one-way (**e**) or two-way (**f**) ANOVA followed by Tukey’s multiple-comparison test. Exact *P* values are denoted in the figure. Source data

**Extended Data Fig. 1:**
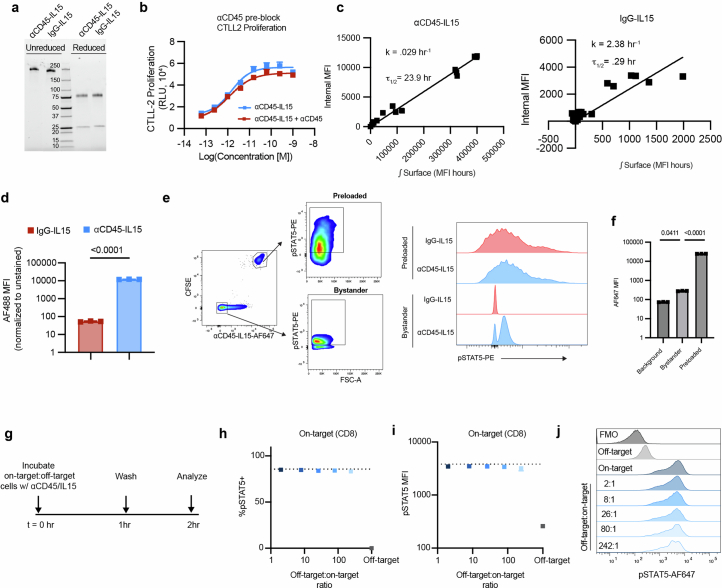
αCD45 immunocytokines show prolonged retention on cell surfaces. **a**, SDS-PAGE of IL15 immunocytokines (representative image of 3 independent protein production batches). Protein ladder molecular weights denoted in kDa. **b**, Luminescence measurement of cell proliferation (n = 3 biological replicates per concentration) following 48 hr incubation with αCD45-IL15 at the indicated concentrations on CTLL-2 cells with or without pre-blocking of CD45 on the reporter cells by incubation with 500 nM αCD45 for 4 hr. **c**, Derivation of internalization rate and half-life for αCD45-IL15 (left) and IgG-IL15 (right) on primary CD8 T cells (n = 3 biological replicates per time point). Surface signal was calculated by fluorescence quenching with an αAF488 antibody. **d**, AF488 signal of CD8^+^ T cells (n = 3 biological replicates) stained for 25 minutes with AF488-labeled αCD45-IL15 or IgG-IL15 for internalization experiment. Unstained background signal was subtracted for analysis. **e**, (Left) gating scheme to define preloaded and bystander cells and their pSTAT5 levels. (Right) Representative pSTAT5 staining (n = 3 biological replicates per condition) for the experimental set-up described in Fig. 1f. **f**, AF647 MFI signal of cells (n = 3 biological replicates per condition) preloaded and bystander cells for the experimental set-up described in Fig. 1f. **g-j**. Activated primary CD8^+^ T cells (on-target) were serially diluted with CD45^-^IL15R^−^ (off-target) cells and incubated with 1 µg per 1 million total cell for 1 hour (n = 3 biological replicates per dilution). Cells were then washed twice and rested for 1 hour before pSTAT5 measurement. **g**, experimental timeline. **h**, Percentage of on-target (CD8^+^) pSTAT5^+^ cells at increasing dilution with CD45^−^IL15R^−^ off-target cells. Dotted line represents undiluted CD8^+^ pSTAT5 signal. **i**, pSTAT5 MFI of on-target (CD8^+^) cells at increasing dilution with CD45^−^IL15R^−^ off-target cells. Dotted line represents undiluted CD8^+^ pSTAT5 signal. **j**, Representative pSTAT5 staining. For all plots, shown are mean ± s.d of the stated replicates. P values were determined by *t*-test or one-way ANOVA followed by Tukey’s multiple comparison test. Exact P values are denoted in the figure. Source data

We have previously shown that antibodies against the ubiquitous leukocyte surface receptor CD45 bind to the surface of T cells and other lymphocytes without triggering internalization^18,19^. We hypothesized that cytokines anchored to cell surfaces using anti-CD45 targeting might exhibit new biological effects relative to the native forms of these cytokines. To test this, we generated αCD45 immunocytokine fusions with IL-15 as a testbed payload. A murine superagonist IL-15 (IL-15 linked to a domain of its receptor α-chain, IL-15Rα_sushi_) was fused carboxy terminally to the heavy chain of an anti-CD45 immunoglobulin (Ig)G2c antibody carrying LALA-PG effector-attenuating mutations (hereafter, αCD45-IL-15; Fig. 1a and Extended Data Fig. 1a)^20^. We also synthesized a nontargeted, size-matched control immunocytokine using an irrelevant fluorescein-specific antibody, referred to hereafter as IgG-IL-15. αCD45-IL-15 bound to plate-bound CD45 and triggered proliferation of IL-15 receptor (IL-15R)-expressing reporter cells (Fig. 1b,c). To assess whether CD45 binding affects the activity of IL-15, we compared the proliferation of reporter cells stimulated by αCD45-IL-15 when CD45 was first pre-blocked using saturating concentrations of αCD45 antibody. αCD45-IL-15 bioactivity was the same whether or not CD45 was available for binding, suggesting that the CD45 epitope targeted by our antibody clone does not generate spatial site resistance that affects cytokine signaling (Extended Data Fig. 1b).

Using dye-labeled constructs and fluorescence quenching, we next assessed the binding and internalization behavior of IL-15 immunocytokines incubated with murine CD8^+^ T cells. Control IgG-IL-15 fusions bound to the cells and were rapidly internalized, whereas αCD45-IL-15 had a cell-surface half-life of 24 h, suggesting that CD45 binding can maintain cytokines on the cell surface irrespective of the rapid internalization behavior of the native cytokine (Fig. 1d and Extended Data Fig. 1c). CD45-targeted IL-15 bound to T cells at far higher levels than IgG-IL-15, as expected given the high expression level of CD45 (Extended Data Fig. 1d). To evaluate whether extended surface retention altered cytokine activity, we pulsed primary CD8^+^ T cells with IL-15 immunocytokines for 1 h, washed to remove unbound cytokine and evaluated downstream signaling. Strikingly, T cells briefly pulsed with αCD45-IL-15 exhibited robust pSTAT5 and CD25 expression levels 24 h later that were almost as high as those of cells incubated continuously in IL-15 (Fig. 1e). By contrast, these markers of IL-15 signaling had all returned to near baseline for cells pulsed with nontargeted IgG-IL-15 (Fig. 1e). We next tested whether CD45-targeted IL-15 can signal both in *cis* and in *trans*. To assess in *trans* signaling, carboxyfluorescein succinimidyl ester (CFSE)-labeled CD8^+^ T cells were preloaded with Alexa Fluor-647 (AF647)-labeled αCD45-IL-15 by pulsing for 1 h with the immunocytokine, then washed and mixed with nonloaded CFSE^−^ bystander CD8^+^ T cells for an additional hour. The presence of just 1 preloaded T cell per 20 total cells led to pSTAT5 induction in most of both the preloaded and the bystander cells, although at a higher level in the former (Fig. 1f and Extended Data Fig. 1e). Signaling to bystander cells occurred with minimal detectable transfer of the labeled cytokine fusion to the bystander cells, suggesting prominent in *trans* signaling of the cytokine on bystander T cells (Extended Data Fig. 1f). By contrast, nontargeted IgG-IL-15 led to a robust pSTAT5 signal in the preloaded population but failed to stimulate bystander cells (Fig. 1f). To confirm that effective *cis* signaling can occur when CD45 is brought into proximity with a cytokine receptor, we serially diluted primary CD8^+^ T cells with excess CD45^−^IL-15R^−^ cells (HEK293F cells) to minimize contact between T cells. HEK-diluted T cells were incubated with αCD45-IL-15 for 1 h, washed, rested for 1 h and then assayed for pSTAT5 expression (Extended Data Fig. 1g). αCD45-IL-15 triggered consistent pSTAT5 signaling in T cells as measured by percentage pSTAT5^+^ or mean fluorescence intensity (MFI), independent of their dilution with CD45^−^IL-15R^−^ cells (Extended Data Fig. 1h–j), suggesting that αCD45-IL-15 is fully capable of *cis* signaling.

**Extended Data Fig. 2:**
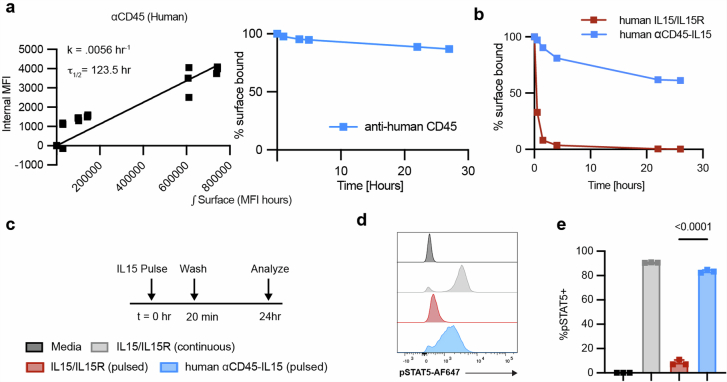
Human αCD45-IL15 is maintained on the cell surface and triggers sustained pSTAT5 induction. **a**, Derivation of internalization rate (left) and internalization kinetics (right) of primary human CD8^+^ T cells incubated with anti-human CD45 labeled with AF488 for stated time points (*n* = 3 biological replicates per time point). Surface signal was calculated by fluorescence quenching with an αAF488 antibody. **b**, Internalization kinetics of human αCD45-IL15 or nontargeted IL15/IL15R labeled with AF488 following binding to primary activated human CD8^+^ T cells *n* = 3 biological replicates per time point. Surface signal was calculated by fluorescence quenching with an αAF488 antibody. **c-e**, Primary human CD8^+^ T cells were pulsed for 20 minutes with human αCD45-IL15 or nontargeted IL15/IL15R (*n* = 3 biological replicates per condition). Cells were washed to remove unbound cytokine, and 24 hours later fixed, permeabilized, and stained for flow cytometry analysis of pSTAT5. **c**, experimental timeline. **d**, representative pSTAT5 histogram. **e**, pSTAT5 + % levels. Data are mean ± s.d. from stated biological replicates. For all plots, shown are mean ± s.d of the stated replicates. P values were determined by *t*-test or one-way ANOVA followed by Tukey’s multiple comparison test. Exact P values are denoted in the figure. Source data

To determine whether these characteristics of CD45-targeted immunocytokines are also observed with human T cells, we first measured the internalization behavior of CD45 on human CD8^+^ T cells. Similar to murine cells, human CD45 exhibited prolonged cell-surface retention with a half-life of >5 d (Extended Data Fig. 2a). We next designed a fully human αCD45-IL-15 molecule composed of a human anti-CD45 IgG1 (clone BC8) fused to a human IL-15/IL-15 receptor (IL-15R) at the heavy chain C terminus. We compared the bioactivity and internalization of human αCD45-IL-15 to an identical IL-15/IL-15R scaffolded as a nontargeted bivalent Fc fusion. Human αCD45-IL-15 exhibited a prolonged cell surface half-life of 28 h on primary human CD8^+^ T cells and, in pulse-chase experiments, sustained pSTAT5 signaling similar to the mouse immunocytokine (Extended Data Fig. 2b–e). In comparison, nontargeted IL-15/IL-15R was rapidly internalized (half-life *τ*_1/2_ = 11 min) and unable to elicit sustained pSTAT5 signaling in the pulse-chase assay.

**Extended Data Fig. 3:**
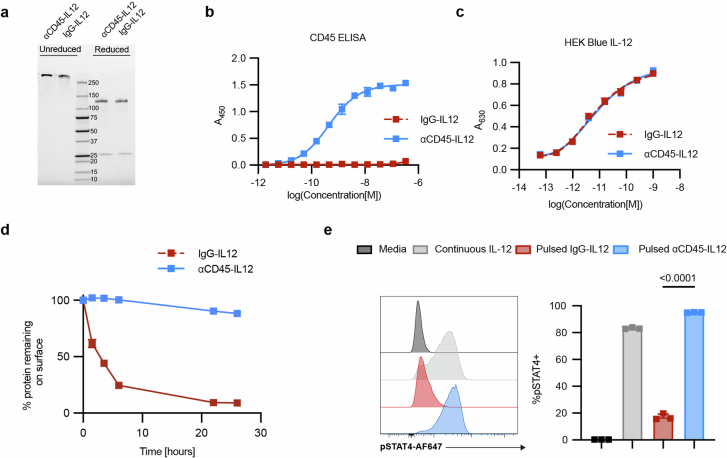
αCD45-IL12 immunocytokines are retained on the cell surface and display extended signaling. **a**, SDS-PAGE of IL12 immunocytokines. Protein ladder molecular weights denoted in kDa. **b**, ELISA absorbance measurement of αCD45-IL12 and IgG-IL12 binding to plate-bound mouse CD45 (*n* = 2 biological replicates). **c**, Absorbance measurement of HEK-Blue-IL12 cells (*n* = 3 biological replicates) following 24 hr incubation with IgG-IL12 or αCD45-IL12 at indicated concentrations. **d**, Internalization kinetics of IgG-IL12 or αCD45-IL12 labeled with AF488 following binding to primary activated CD8^+^ T cells (*n* = 3 biological replicates). Surface signal was calculated by fluorescence quenching with an αAF488 antibody; **e**, Primary CD8^+^ T cells (*n* = 3 biological replicates) were pulsed for with IL-12 immunocytokine fusions for 20 minutes. Cells were washed to remove unbound cytokine, and 24 hours later fixed, permeabilized, and stained for flow cytometry analysis of pSTAT4. In **b-e**, data are mean ± s.d. from stated replicates. P values were determined by one-way ANOVA followed by Tukey’s multiple comparison test. Exact P values are denoted in the figure. Source data

To test whether cell-surface retention and prolonged signaling were a general phenomenon for αCD45–cytokine fusions, we also generated αCD45-IL-12, a fusion of a single chain IL-12p70 with αCD45. This IL-12 immunocytokine was able to bind CD45 with no loss of cytokine bioactivity and exhibited prolonged surface retention and signaling compared with IgG-IL-12, as measured by prolonged pSTAT4 levels (Extended Data Fig. 3a–e). Thus, although CD45’s phosphatase properties have been previously employed to deactivate nearby tethered receptors^21^, CD45-targeted antibody–cytokine fusions, as designed in the present study, exhibit sustained and potent signaling.

### Local αCD45-IL-15 persists at the tumor and TDLNs

**Fig. 2:**
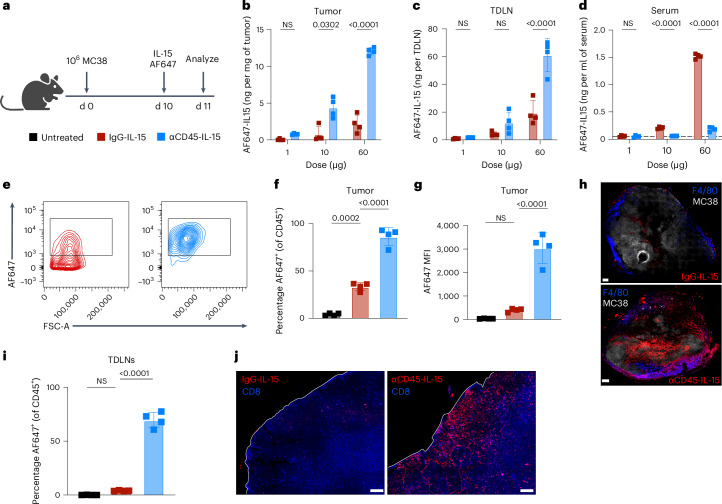
Locally delivered αCD45-IL-15 is retained in the tumor and TDLNs with negligible systemic exposure. **a**, Study diagram. **b**–**d**, C57BL/6J mice (*n* = 4 mice per group) were inoculated with 10^6^ MC38 cells in the flank and 10 d later administered AF647-labeled αCD45-IL-15 or IgG-IL-15 at a 1-, 10- or 60-µg dose. After 24 h, organs were harvested, lysed and analyzed for cytokine signal by fluorescence spectroscopy measurements. Concentrations were calculated using a standard curve. The dashed lines represent the limit of detection. **b**, Tumor concentrations of dosed immunocytokines. **c**, TDLN concentrations of dosed immunocytokines. **d**, Serum concentrations of dosed immunocytokines. **e**–**j**. C57BL/6J mice (*n* = 4 mice per group) were inoculated with 10^6^ MC38 (**e**–**g** and **i**) or MC38-ZsGreen (**h** and **j**) cells in the flank and, 10 d later, administered AF647-labeled αCD45-IL-15 or IgG-IL-15 at a 10-µg dose i.t., followed by analysis of cytokine biodistribution 24 h later by flow cytometry and microscopy. **e**, Representative contour plots of antibody–cytokine fusion binding to CD45^+^ TILs. **f**, Frequencies of AF647^+^ TILs. **g**, AF647 MFI on TILs. **h**, Representative confocal images of tumors. Scale bar, 200 μm. **i**, AF647 labeling on TDLN immune cells. **j**, Representative histological images of TDLNs. Scale bar, 100 μm. Shown are mean ± s.d. from *n* = 4 mice per group. *P* values were determined by two-way (**b**–**d**) or one-way (**f**, **g** and **i**) ANOVA followed by Tukey’s multiple-comparison test. Exact *P* values are denoted in the figure; NS, not significant (*P* > 0.05). Source data

**Extended Data Fig. 4:**
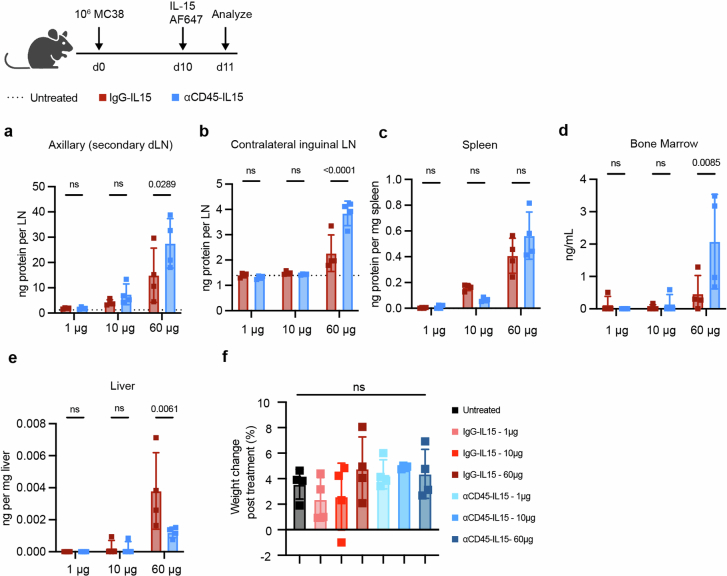
Intratumoral αCD45-IL15 drains from the tumor through the TDLN in a dose-dependent manner. C57BL/6 J mice (n = 4 mice/group) were inoculated with 10^6^ MC38 cells in the flank, and 10 days later administered AF647-labeled αCD45-IL15 or IgG-IL15 *at* 1, 10, or 60 µg doses. 24 hours later, organs were harvested, lysed, and analyzed for cytokine signal by fluorescence spectroscopy measurements. Concentrations were calculated using a standard curve. Dotted lines represent limit of detection. **a**, axillary LN (secondary draining) concentrations of dosed immunocytokines. **b**, contralateral inguinal (non-draining) LN concentrations of dosed immunocytokines. **c**, spleen concentrations of dosed immunocytokines. **d**, femur bone marrow concentrations of dosed immunocytokines. **e**, liver concentrations of dosed immunocytokines. **f**, weight change after treatment. Statistical comparison is to the untreated group. For all plots, shown are mean ± s.d of the stated replicates. P values were determined by one-way, or two-way ANOVA followed by Tukey’s multiple comparison test. Exact P values are denoted in the figure; “ns” = not significant (*P* > 0.05). Source data

We next sought to understand how the biodistribution of CD45–cytokine fusions is impacted by the dose administered. Mice bearing established MC38 tumors received an intratumoral injection of AF647-labeled αCD45-IL-15 or IgG-IL-15 at a 1-, 10- or 60-µg dose. After 24 h, we harvested tumors, inguinal TDLNs, axillary LNs (secondary draining LNs), nondraining contralateral LNs, spleen, liver and bone marrow for measurement of total cytokine fusion present by fluorescence analysis of tissue lysates (Fig. 2a). This analysis revealed a dose-dependent distribution pattern for αCD45–cytokines: At 1 µg, the protein was entirely retained in the tumor (Fig. 2b–d and Extended Data Fig. 4a–e). At 10 µg, αCD45-IL-15 was detected in the tumor and draining inguinal and axillary LNs, but was undetectable in contralateral LNs, blood, liver and bone marrow, and was measured at very low levels in the spleen (Fig. 2b–d and Extended Data Fig. 4a–e). By contrast, IgG-IL-15 at this dose was not retained in the tumor and showed dissemination into the blood and accumulation in the spleen (Fig. 2b–d and Extended Data Fig. 4a–e). Finally, at a substantially higher dose of 60 µg, we measured spillover from on-target tissues (tumor, TDLNs) into off-target tissues (blood, spleen, contralateral LNs) for both αCD45-IL-15 and IgG-IL-15 (Fig. 2b–d and Extended Data Fig. 4a–e). Notably, across all these doses, treated animals did not experience any significant weight loss (Extended Data Fig. 4f). Thus, at a dose of 10 µg, intratumoral administration of αCD45–cytokines primarily leads to uptake in the tumor and TDLNs, while sparing systemic tissues.

**Extended Data Fig. 5:**
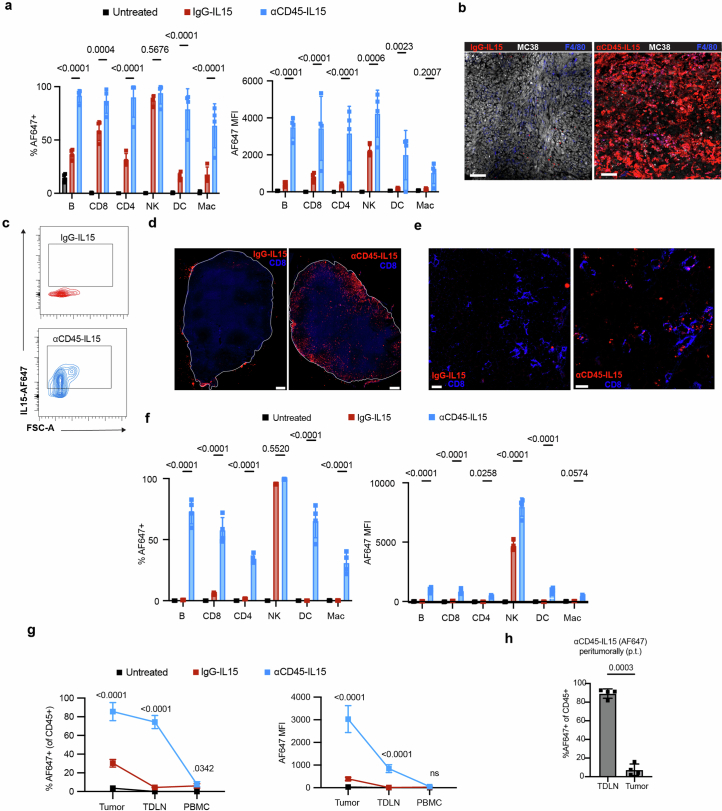
αCD45-IL15 displays altered biodistribution compared to non-targeted IgG-IL15. **a-g**, AF647-labeled αCD45-IL15 or IgG-IL15 was dosed (10 µg) intratumorally in established MC38 tumors 24 hours prior to flow cytometry analysis (n = 4 mice/group). Shown are **a**, Tumor cell-type specific biodistribution by percentage (left) or MFI (right). **b**, Representative tumor images from the experiment described in Fig. 2a. Scale bar, 50 µm. **c**, Representative contour plot of AF647 signal on TDLN CD45^+^ cells. **d**, Representative TDLN images from the experiment described in Fig. 2a. Scale bar, 200 µm. **e**, Representative TDLN images from the experiment described in Fig. 2a. Scale bar, 10 µm. **f**, TDLN cell-type specific biodistribution by percentage (left) or MFI (right). **g**, Compartment specific biodistribution by MFI (left) or percentage (right). **h**, AF647-labeled αCD45-IL15 was dosed (10 µg) peritumorally (s.c. at the tail-base) 24 hours prior to flow cytometry analysis (n = 4 mice/group). For all plots, shown are mean ± s.d of the stated replicates. P values were determined by two-sided *t*-test, one-way, or two-way ANOVA followed by Tukey’s multiple comparison test. Exact P values are denoted in the figure; “ns” = not significant (P > 0.05). Source data

Based on these biodistribution characteristics, we next assessed binding of the immunocytokines to leukocytes in the tumor and TDLNs by flow cytometry and microscopy following the experimental set-up of Fig. 2a. Injection of 10 µg of AF647-labeled αCD45-IL-15 led to robust labeling of most CD45^+^ cells in the tumor (Fig. 2e–g). By contrast, the nontargeted control cytokine showed association with only ~40% of intratumoral CD45^+^ cells and at much lower MFI levels than the αCD45 fusion (Fig. 2e–g). αCD45-IL-15 bound effectively to all common intratumoral immune subsets whereas IgG-IL-15 mostly associated with natural killer (NK) cells, probably as a result of their high IL-2Rβγ expression (Extended Data Fig. 5a). Confocal microscopy revealed a broad distribution of αCD45-IL-15 throughout injected tumors, whereas IgG-IL-15 exposure was sparse and isolated to narrow regions (Fig. 2h and Extended Data Fig. 5b). This dose also led to labeling of ~75% of CD45^+^ cells in the TDLNs by αCD45-IL-15, whereas IgG-IL-15 uptake was not statistically different from untreated controls (Fig. 2i and Extended Data Fig. 5c). Imaging of histological sections revealed efficient drainage of labeled αCD45-IL-15 throughout the TDLNs (Fig. 2j and Extended Data Fig. 5d,e). Flow cytometry analysis of the TDLNs again revealed NK cells as the only cellular subset with substantial uptake of IgG-IL-15, whereas αCD45-IL15 was bound to T cells, B cells, macrophages and dendritic cells (Extended Data Fig. 5f). Importantly, at this dose, there was minimal association with peripheral blood mononuclear cells (PBMCs; Extended Data Fig. 5g). Intratumoral administration was required for simultaneous tumor and LN labeling, as peritumorally delivered αCD45-IL-15 efficiently labeled the TDLNs but failed to accumulate in the tumor (Extended Data Fig. 5h). Thus, CD45 targeting increased localization of the cytokine on immune cells in both tumors and TDLNs, while avoiding systemic exposure when doses were selected to not exceed the binding capacity of leukocyte surface CD45 in these tissues.

### αCD45–cytokine therapy eradicates treated tumors

Next, we evaluated the impact of this altered signaling and biodistribution on the therapeutic potential of a cytokine therapy employing both αCD45-IL-15 and αCD45-IL-12. We first treated established tumors 30–35 mm^2^ in size with a single intratumoral injection of 1 µg αCD45-IL-12 followed by one injection of 10 µg αCD45-IL-15 several days later. Our rationale for this sequencing was to use the IL-12 immunocytokine to inflame the tumor and promote an initial wave of tumor antigen release by pre-existing or newly recruited tumor-infiltrating leukocytes (TILs)^11^, followed by amplification of newly primed, tumor-specific T cells in the TDLNs by the IL-15 immunocytokine several days later^22,23^. The selected doses were informed by findings from the biodistribution experiments and aimed to retain IL-12 at the tumor but deliver IL-15 to both the tumor and the TDLNs. Hereafter, we refer to this sequential αCD45-IL-12/αCD45-IL-15 regimen as αCD45-Cyt; the untargeted cytokine control therapy consisting of IgG-IL-12 followed by IgG-IL-15 is referred to as IgG-Cyt.

**Fig. 3:**
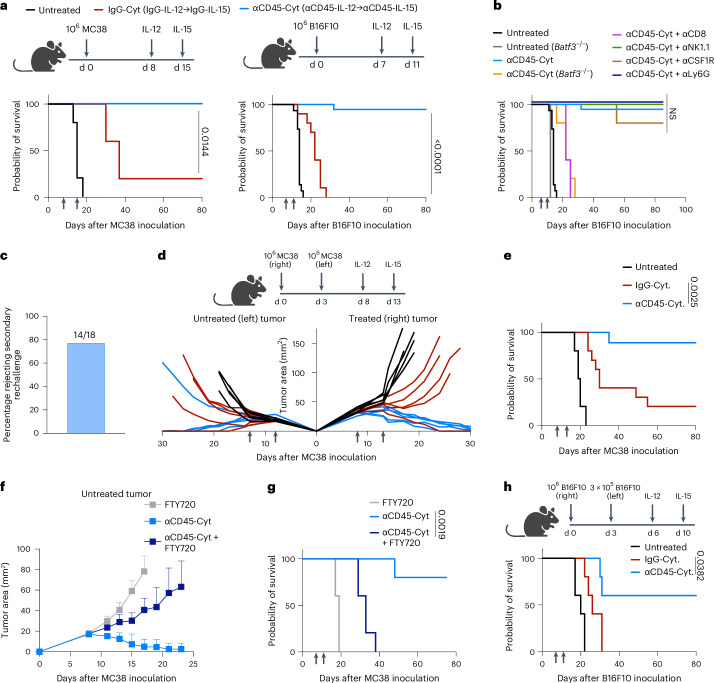
αCD45–cytokine therapy eradicates treated tumors and elicits robust systemic responses. **a**, Overall survival of C57BL/6J mice (MC38: *n* *=* 5 per group; B16F10: *n* *=* 10 in untreated cohort, *n* *=* 10 in IgG-Cyt cohort, *n* *=* 19 in αCD45-Cyt cohort across two to five independent experiments) inoculated with 10^6^ tumor cells and treated with 1 µg of αCD45-IL-12 and 10 µg of αCD45-IL-15 (referred to as αCD45-Cyt) or treated with 1 µg of IgG-IL-12 and 10 µg of IgG-IL-15 (referred to as IgG-Cyt) as shown on the experimental timelines. d, day. Statistical comparison calculated by log(rank) (Mantel–Cox) test shown between IgG-Cyt and αCD45-Cyt groups. **b**, Overall survival of C57BL/6J mice (*n* *=* 5/group) bearing B16F10 tumors treated with αCD45-Cyt therapy in combination with depleting antibodies or performed in knockout mice as shown. **c**, Overall survival of C57BL/6J mice cured of B16F10 tumors rechallenged with 10^5^ B16F10 cells 100 d after the initial challenge. **d**, C57BL/6J mice bearing MC38 tumors on both flanks treated with 1 µg of IL-12 and 10 µg of IL-15 in the right tumor only at the indicated times (*n* *=* 10 mice in untreated cohort, *n* *=* 10 in IgG-Cyt cohort, *n* *=* 9 in αCD45-Cyt cohort across two independent experiments). Individual tumor growth curves from one representative experiment are shown. **e**, Overall survival from experiment shown in **d**. **f**, C57BL/6J mice bearing bilateral MC38 tumors (*n* *=* 5 mice in all treatment groups) treated with αCD45-Cyt therapy in the presence of FTY720 starting on day 5. The mean tumor growth curve ± s.d. is shown. **g**, Overall survival from experiment shown in **f**. **h**, Overall survival of C57BL/6J mice bearing contralateral B16F10 tumors treated with 1 µg of IL-12 and 10 µg of IL-15 as shown (*n* *=* 5 mice per group). *P* values were determined by log(rank) (Mantel–Cox) test from stated replicates. Exact *P* values are denoted in the figure. For all the plots, the arrows indicate treatment. Source data

**Extended Data Fig. 6:**
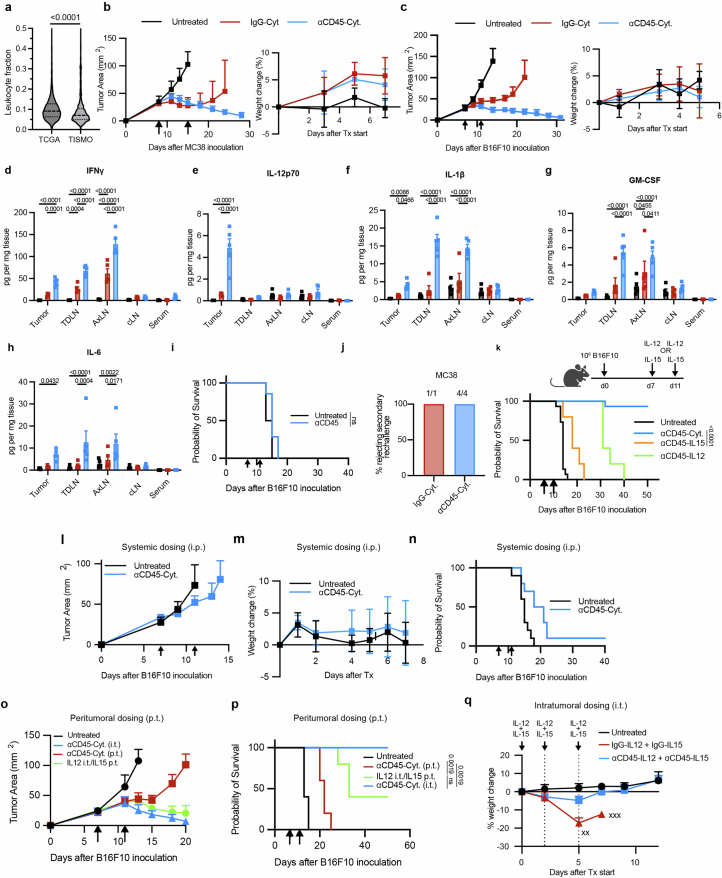
αCD45-Cytokine therapy eradicates injected tumors. **a**, Leukocyte fraction calculated from EPIC RNA-seq analysis across all TCGA entries (n = 11070) or TISMO mouse entries (*n* = 1518). **b**, Tumor growth curves (left) and weights (right) of C57BL/6 J mice (n = 5/group) inoculated with 10^6^ MC38 tumor cells and treated with IgG-Cyt therapy or αCD45-Cyt therapy as described in Fig. 3a. **c**, Representative tumor growth curves (left) and weights (right) of C57BL/6 J mice (n = 5 mice /group representative of 3-4 independent experiments) inoculated with 10^6^ B16F10 tumor cells and treated with IgG-Cyt therapy or αCD45-Cyt therapy as described in Fig. 3a. **d-h**. C57BL/6 J mice (n = 5 in untreated group, n = 5 in IgG-Cyt group, n = 5 in αCD45-Cyt group) inoculated with 10^6^ B16F10 tumor cells were treated with IgG-Cyt or αCD45-Cyt therapy beginning on d7. One day after completing therapy, tumors, TDLNs, axillary draining lymph nodes (AxLN), non-draining contralateral lymph nodes (cLN), and serum were harvested, lysed, and analyzed for cytokine expression. Shown are the analyte concentrations in pg per mg of tissue for **d**, IFNg **e**, IL-12p70 **f**, IL-1b **g**, GM-CSF and **h**, IL-6. **i**, Overall survival of C57BL/6 J mice (n = 6 in PBS cohort, n = 7 in αCD45 group) bearing B16F10 tumors treated with αCD45 (in the absence of cytokine) on an identical regimen and molar matched dose to that described in Fig. 3a. **j**, Overall survival of C57BL/6 J mice cured of MC38 tumors rechallenged with 10^5^ MC38 cells 100 days after the initial challenge. **k**, Overall survival of C57BL/6 J mice (n = 5/group) inoculated with 10^6^ B16F10 tumor cells and treated with IL-12 immunocytokine monotherapy (1 µg dose) or IL-15 immunocytokine monotherapy (10 µg dose) as shown on the diagram. **l**, Tumor growth curves of C57BL/6 J mice (n = 5/group representative of 2 independent experiments) inoculated with 10^6^ B16F10 cells and treated systemically (i.p.) with αCD45-Cyt therapy with the same doses and schedule as in Fig. 3a. **m**, Weights of mice shown in l. **n**, Overall survival of mice shown in l across 2 independent experiments (*n* = 10 in all groups). **o**, Tumor growth curves of C57BL/6 J mice (*n* = 5 in all treatment groups) inoculated with 10^6^ B16F10 cells and treated with the full αCD45-Cyt intratumorally, peritumorally, or the IL-12 immunocytokine given intratumorally and the IL-15 immunocytokine given peritumorally, as described in the text. **p**, Overall survival of the mice shown in o. **q**, Weight of mice (n = 5/group) inoculated with 10^6^ MC38 tumor cells and simultaneously treated with 4 µg IL-12 and 20 µg of IL-15 intratumorally on days marked with dotted lines and arrows. “x” denotes euthanasia due to poor body condition or weight-related criteria. Shown are mean ± s.d. from stated replicates. P values were determined by two-sided *t*-test, log-rank (Mantel-Cox), or two-way ANOVA followed by Tukey’s multiple comparison test. Exact P values are denoted in the figure; “ns” = not significant (P > 0.05). Source data

We tested this therapeutic regimen in two syngeneic tumor models, MC38 colorectal carcinoma, which is partially responsive to checkpoint blockade immunotherapy, and B16F10, an aggressive and poorly responsive melanoma model (Fig. 3a). Notably, mouse syngeneic tumor models have a range of CD45^+^ leukocyte fractions that overlap with those reported for human tumors (Extended Data Fig. 6a)^24^. Injection of control IgG-Cyt elicited an extension in survival, but few (MC38) or no (B16F10) animals were long-term survivors (Fig. 3a and Extended Data Fig. 6b,c). By contrast, 100% (MC38) or >90% (B16F10) of tumors treated with αCD45-Cyt therapy were rejected and animals exhibited no signs of cytokine toxicity, as assessed via weight loss (Fig. 3a and Extended Data Fig. 6b,c).

To gain insight into the mechanism of action, we first assessed the extent and localization of cytokine induction after αCD45-Cyt treatment. Mice bearing B16F10 tumors were treated with αCD45-Cyt therapy as in Fig. 3a and, 24 h after the IL-15 dose, we analyzed the expression of inflammatory cytokines interferon (IFN)γ, IL-12p70, IL-1β, granulocyte–macrophage colony-stimulating factor and IL-6 in the tumor, inguinal TDLN, axillary LN, contralateral LN and blood. Cytokine induction was primarily limited to the on-target tissues (tumor and TDLNs), whereas cytokine levels in the blood and activation within contralateral nondraining LNs were not significantly elevated above baseline, functionally highlighting the localized nature of αCD45-Cyt therapy (Extended Data Fig. 6d–h). Potential modulation of the immune response by CD45 engagement did not contribute to efficacy, because treatment with αCD45 in the absence of cytokines provided no tumor control (Extended Data Fig. 6i). We assessed the mechanism of tumor rejection by αCD45-Cyt therapy through antibody depletions and knockout mice: αCD45-Cyt therapy entirely depended on CD8^+^ T cells and *batf3*^*+*^ dendritic cells, consistent with a critical role for tumor antigen crosspresentation (Fig. 3b). NK cells, neutrophils and macrophages were each dispensable for antitumor efficacy (Fig. 3b). Mice cured of MC38 and B16F10 tumors by αCD45-Cyt therapy displayed robust immune memory on secondary tumor rechallenge (Fig. 3c and Extended Data Fig. 6j). Use of both cytokines in the treatment was required, because αCD45-IL-15 or αCD45-IL-12 monotherapy using the same treatment schedule elicited no long-term survivors (Extended Data Fig. 6k).

To study the importance of cytokine localization to the tumor and TDLNs, we varied the route of administration of αCD45-Cyt therapy. The same doses of αCD45-Cyt treatment administered systemically (intraperitoneally (i.p.)) led to a complete loss of efficacy, confirming the importance of robust tumor and TDLN exposure for activity (Extended Data Fig. 6l–n). Administration of αCD45-Cyt therapy peritumorally, which predominantly acts on the TDLNs while sparing the tumor (Extended Data Fig. 5h), completely ablated efficacy (Extended Data Fig. 6o,p). Intratumoral αCD45-IL-12, followed by peritumorally delivered αCD45-IL-15, displayed comparable early tumor control to the full intratumoral paradigm, but most of these mice (three of five) eventually died, suggesting that optimal long-term tumor control required both doses to be administered i.t. (Extended Data Fig. 6o,p). Finally, to assess the safety of the treatment, we assessed weight loss in mice simultaneously treated with a combination of IL-12 and IL-15 intratumorally at doses previously reported to induce treatment-related weight loss^10^. When dosed in combination, 4 µg of untargeted IgG-IL-12 and 20 µg of IgG-IL-15 led to severe weight loss and poor body conditions requiring euthanasia across the entire cohort. In contrast, the same doses of αCD45-IL-12 + αCD45-IL-15 triggered only a minor transient weight loss from which the mice entirely recovered (Extended Data Fig. 6q).

### αCD45–cytokine therapy elicits systemic immunity

**Extended Data Fig. 7:**
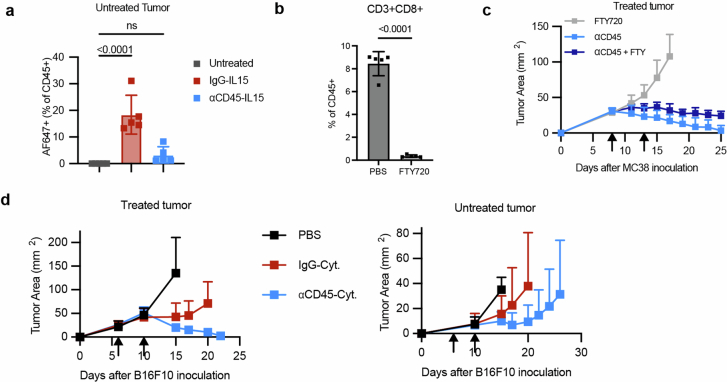
αCD45-Cytokine therapy drives abscopal effects. **a**, C57BL/6 J mice (n = 5/group) were inoculated with bilateral MC38 tumors as described and dosed with a therapeutic dose of AF647-labeled IL-15 immunocytokine. Shown are AF647 levels on immune cells at the contralateral tumor 24 hours after administration of IL-15. **b**, C57BL/6 J mice (n = 5/group) were dosed i.p. with 1.5 mg/kg FTY720. Two days later, PBMCs were collected and shown is the CD8^+^ T cell frequency. **c**, C57BL/6 J mice (n = 5 mice in all treatment groups) bearing bilateral MC38 tumors were treated with αCD45-Cyt therapy and 1.5 mg/kg FTY720 i.p. starting on d5 every other day. Shown is the average tumor growth curve for the treated tumor. **d**, C57BL/6 J mice (n = 5 mice in all treatment groups) bearing bilateral B16F10 tumors were treated with IgG-Cyt or αCD45-Cyt therapy as described. Shown are tumor growth curves for the treated tumor (left) and contralateral tumor (right). Shown are mean ± s.d. P values were determined by two-sided *t*-test or one-way ANOVA followed by Tukey’s multiple comparison test. Exact P values are denoted in the figure; “ns” = not significant (P > 0.05). Source data

The successful translation of i.t. administered immunotherapies will depend on their ability to eliminate lesions that are not directly treated. To evaluate abscopal immune responses elicited by αCD45-Cyt therapy, MC38 tumors were inoculated on the opposite flanks of mice followed by treatment of only the right-flank tumor with αCD45-Cyt therapy (Fig. 3d). Strikingly, this led to 90% of mice rejecting both the treated and untreated tumors and becoming long-term survivors, whereas only 20% of animals receiving IgG-Cyt therapy eliminated both tumors (Fig. 3d,e). These results were obtained despite the fact that tracking of fluorescently labeled constructs showed that locally delivered IgG-Cyt readily diffused out of the treated lesion and accumulated in the distal site, whereas no statistically significant accumulation of αCD45-Cyt was measured at the untreated tumor (Extended Data Fig. 7a). To determine whether regression of distal tumors relied on lymphocyte trafficking, we treated mice bearing bilateral MC38 tumors in the presence of FTY720 to block lymphocyte exit from lymphoid tissues (Extended Data Fig. 7b). FTY720 treatment ablated the ability of αCD45-Cyt to control the untreated left-flank tumor and led to no long-term survivors, suggesting that rejection of untreated lesions is driven by lymphocyte migration (Fig. 3f,g). αCD45-Cyt therapy still triggered regression of the treated tumor in the presence of FTY720, but no tumors had been cured at the time of euthanasia owing to outgrowth of the untreated distal lesion (Extended Data Fig. 7c). To further stress test this treatment, we tested abscopal responses in the aggressive B16F10 model. Although IgG-Cyt treatment led to some delay of treated tumors, all untreated tumors progressed (Fig. 3h and Extended Data Fig. 7d). By contrast, αCD45-Cyt therapy led to primary tumor regression in all mice and only two of five untreated tumors escaped (Fig. 3h and Extended Data Fig. 7d).

**Extended Data Fig. 8:**
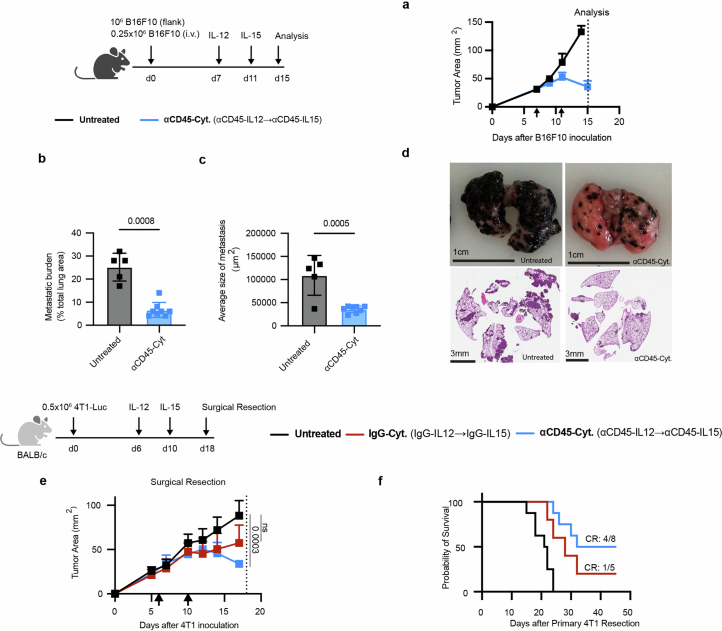
αCD45-Cyt therapy triggers systemic anti-tumor immunity. **a-d**, C57BL/6 J (n = 5 in untreated cohort, n = 8 in αCD45-Cyt cohort) were simultaneously inoculated with 10^6^ B16F10 cells s.c. and 2×10^5^ cells retro-orbitally (r.o.) to develop lung metastases. Mice were treated with 1 μg αCD45-IL12 immunocytokine on day 7 and 10 μg αCD45-IL15 on day 11 (αCD45-Cyt). On d15, animals were euthanized and tumor burden was analyzed. **a**, Tumor growth curves with arrows denoting treatment. Dashed line denotes analysis timeline. **b**, Metastatic lung burden shown as percentage of lung tissue after αCD45-Cyt treatment. **c**, Average metastasis size after αCD45-Cyt treatment. **d**, Representative images of untreated (top left) or αCD45-Cyt treated (top right) lungs. Representative histology of untreated (bottom left) or αCD45-Cyt treated (bottom right) lungs. Shown are mean ± s.d. For metastatic burden, P values were determined by Mann-Whitney non-parametric test. For average metastasis size, P values were determined by two-sided *t*-test. **e-f**, BALB/c mice (*n* = 8 animals/group for untreated, *n* = 5 for IgG-Cyt, *n* = 8 for αCD45-Cyt) were inoculated with 0.5×10^6^ 4T1^−^Luc cells in the mammary fat pad, and 6 days later, tumors were treated with IgG-Cyt or αCD45-Cyt therapy. On day 18, primary tumors were resected. Shown are **e**, experimental timeline and **f**, overall survival. Data are mean ± s.d. from individual mice. P values were determined by two-way ANOVA followed by Tukey’s multiple comparison test at day 17. Exact P values are denoted in the figure; “ns” = not significant (P > 0.05). Source data

Next, we assessed the ability of αCD45-Cyt therapy to eliminate metastases through two models: first, we treated mice that had been injected with 2 × 10^5^ B16F10 cells intravenously in addition to flank tumor inoculation, giving rise to lung metastases (Extended Data Fig. 8a–d). Once untreated mice had reached euthanasia criteria due to flank tumor outgrowth (day 15), we analyzed lung metastases in all groups. Regression of the treated primary tumor was in progress for αCD45-Cyt therapy at this time point (Extended Data Fig. 8a–d). We also found a dramatic reduction in total metastatic tumor burden and average metastatic size (Extended Data Fig. 8a–d). Finally, we evaluated αCD45-Cyt therapy in the triple-negative breast cancer 4T1 model using a neoadjuvant treatment approach. We inoculated 0.5 × 10^6^ 4T1-Luc cells orthotopically into the mammary fat pad of BALB/c mice and, beginning at day 6, treated with IgG-Cyt or αCD45-Cyt therapy (Extended Data Fig. 8e,f). When the untreated mice had reached the euthanasia criteria (day 18), we surgically resected the primary tumors and monitored for survival and relapse. At the time of resection, only αCD45-Cyt therapy had significantly slowed tumor growth (Extended Data Fig. 8e). Untreated animals experienced rapid outgrowth of lung metastases and none survived (Extended Data Fig. 8f). Of mice treated with αCD45-Cyt therapy, 50% were protected from lung metastases, whereas only 20% of mice treated with control IgG-Cyt therapy survived long term. Altogether, we found that αCD45-Cyt therapy can prime a robust systemic immune response after localized treatment that is effective against seeded and spontaneous metastases at a tissue site distinct from the primary tumor.

### Treated TDLN T cells have an acute antiviral signature

**Fig. 4:**
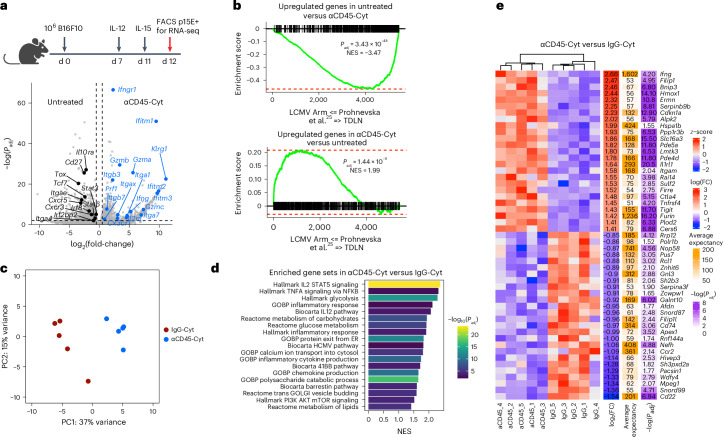
αCD45-Cyt therapy induces antiviral-like signature in TDLN tumor-specific T cells. CD8^+^ T cells specific for the immunodominant p15E retroviral antigen were sorted from TDLNs isolated from C57BL/6J mice bearing B16F10 tumors (*n* = 4 mice in PBS cohort) treated with αCD45-Cyt (*n* = 5 mice) or IgG-Cyt (*n* = 5 mice) therapy for downstream RNA-seq. **a**, Volcano plot of DEGs between untreated and αCD45-Cyt with selected genes labeled. **b**, GSEA using the DEGs between untreated and αCD45-Cyt, compared with T cell gene signatures from LCMV Arm infection or TDLNs from Prokhnevska et al.^25^ (GEO accession no. GSE216731). The enrichment score is plotted. Adjusted *P* (*P*_adj_) values and normalized enrichment scores (NESs) are labeled. **c**, PCA of IgG-Cyt- and αCD45-Cyt-treated samples. **d**, Enriched pathways in αCD45-Cyt samples compared with IgG-Cyt samples. **e**, Top 25 DEGs between αCD45-Cyt samples and IgG-Cyt samples. FC, fold-change. Statistical significance was determined using a two-sided Wald’s test with Benjamini–Hochberg post-hoc correction (**a** and **e**) or one-sided permutation test (**b** and **d**). Source data

We next sought to understand how localized αCD45-Cyt treatment elicited such potent systemic immunity, focusing on the CD8^+^ T cells required for efficacy. A recent report demonstrating the spatial segregation of T cell priming and activation between the TDLNs and tumor^25^, respectively, motivated us to profile cells from the TDLNs. We treated B16F10 tumors with αCD45-Cyt or IgG-Cyt therapy and, 1 d after dosing of the IL-15 immunocytokine, sorted CD8^+^ T cells specific for the immunodominant p15E endogenous retroviral antigen expressed by B16F10 tumors^26^ via peptide–major histocompatibility complex (MHC) tetramer staining for downstream RNA sequencing (RNA-seq). αCD45-Cyt therapy dramatically reprogrammed the CD8^+^ T cell response, leading to significant upregulation of 1,726 genes and downregulation of 1,279 genes in comparison to the untreated condition (Fig. 4a). Many upregulated genes were associated with activation and effector function (*IL2ra*, *Gzmb*, *Klrg1* and *Prf1*) as well as IFN signaling (*Ifng*, *Ifngr1* and *Ifitm1*), suggesting an ability of αCD45-Cyt therapy to promote activation and effector differentiation of T cells within the TDLN (Fig. 4a). Concurrently, genes related to LN trafficking (*Cxcr3* and *Cxcr5*) as well as stemness (*Tcf7*) were downregulated (Fig. 4a). Despite this highly differentiated phenotype, we saw significant downregulation of *Tox*, a transcription factor responsible for initiating epigenetic exhaustion programs in CD8^+^ T cells.

**Extended Data Fig. 9:**
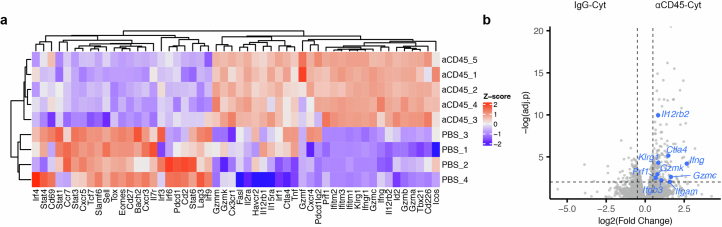
αCD45-Cyt therapy enhances activation of the tumor-specific TDLN compartment. C57BL/6 J mice bearing B16F10 tumors (*n* = 4 mice in PBS cohort) treated with αCD45-Cyt (*n* = 5 mice) or IgG-Cyt (*n* = 5 mice) therapy were sorted for CD8^+^ T cells specific for the immunodominant p15E retroviral antigen for downstream RNA-seq. **a**, Differential gene expression of select inflammatory and activation markers between untreated and αCD45-Cyt treated mice. **b**, Volcano plot of differentially expressed genes between IgG-Cyt and αCD45-Cyt with selected genes labeled. Statistical significance was determined using a two-sided Wald test with Benjamini-Hochberg post hoc correction. Source data

Unlike the endogenous antitumor response, effective virus-specific T cell responses, such as after acute lymphocytic choriomeningitis virus Armstrong (LCMV Arm) infection, are accompanied by high expression of inflammatory cytokines and differentiation markers in responding LNs^25^. Given the efficacy of αCD45-Cyt therapy, we performed gene set enrichment analysis (GSEA) to probe whether αCD45-Cyt therapy induces an ‘antiviral-like’ signature within the tumor-reactive TDLN CD8^+^ compartment. We assessed the untreated and αCD45-Cyt-treated transcriptomes of tumor-specific CD8^+^ T cells for enrichment of a previously reported tumor (‘TDLN’) or acute viral (‘LCMV Arm’) transcriptional response (Fig. 4b). Transcriptional profiles of CD8^+^ T cells from untreated TDLNs closely matched the previously reported endogenous antitumor response, broadly characterized by a lack of effector molecules and activation markers (Fig. 4b and Extended Data Fig. 9a). In contrast, αCD45-Cyt therapy elicited a signature highly enriched in genes associated with the canonical antiviral response, suggesting that the prolonged cytokine exposure within TDLNs induced by αCD45-Cyt triggers an immune response mirroring effective antiviral T cell priming (Fig. 4b and Extended Data Fig. 9a). We next characterized differences in the transcriptional response of αCD45-Cyt treatment versus control IgG-Cyt therapy. Principal component analysis (PCA) showed that IgG-Cyt and αCD45-Cyt transcriptomes clustered separately (Fig. 4c). Pathway analysis comparing the two treatment conditions revealed a striking upregulation of hallmark STAT5 signaling triggered by αCD45-Cyt therapy, probably driven by the extended signaling of CD45-bound IL-15, as shown in Fig. 1e (Fig. 4d). Other upregulated genes belonged to pathways involving inflammatory responses, cytokine signaling and cell-cycle metabolism (Fig. 4d). Finally, we examined the top 20 differentially expressed genes (DEGs) between αCD45-Cyt and IgG-Cyt therapy and found *Ifng* expression as the most differentially upregulated, along with other metabolism and inflammation genes (Fig. 4e and Extended Data Fig. 9b). Altogether, these data are indicative of a highly activated and proliferative TDLN compartment after αCD45-Cyt therapy.

### αCD45 immunocytokines generate optimal effectors

**Fig. 5:**
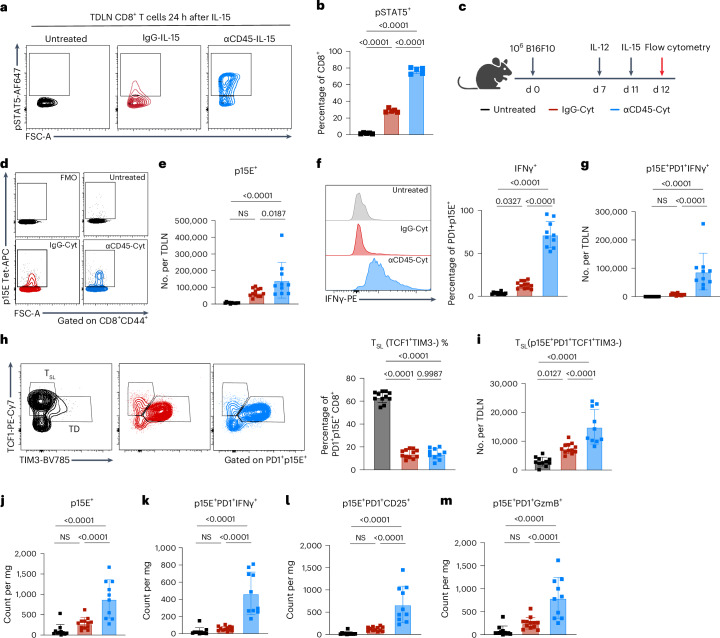
αCD45–cytokine therapy reprograms tumor-specific TDLNs and tumor CD8^+^ T cells. **a**,**b**, C57BL/6J mice (*n* *=* 5 per group) inoculated with 10^6^ B16F10 cells were treated on day 7 with 10 μg of IL-15 immunocytokine. After 24 h, LNs were fixed, permeabilized and stained for pSTAT5. **a**, Representative pSTAT5 contour plots after IL-15 treatment, previously gated on live CD3^+^CD8^+^ cells. **b**, Summary data for pSTAT5 induction shown in **a**. **c**–**m**, C57BL/6J mice (*n* *=* 11 in untreated cohort, *n* *=* 12 in IgG-Cyt cohort, *n* *=* 10 in αCD45-Cyt cohort across two independent studies) inoculated with 10^6^ B16F10 tumor cells treated with IgG-Cyt or αCD45-Cyt therapy starting on day 7 (**c**). LNs and tumors were harvested 24 h after completion of therapy. **d**, Representative contour plots and gating for p15E tetramer^+^ cells in the TDLNs, previously gated on live CD8^+^CD44^+^ cells. **e**, Treatment effects on p15E^+^ tumor-reactive cells in TDLNs. **f**, Left, sample histogram of IFNγ expression; right, TDLN frequency of tumor-specific cells expressing IFNγ^+^. **g**, TDLN counts of IFNγ^+^PD-1^+^p15E^+^ T cells. **h**, Left, T_SL_ versus TD sample gating; right, TDLN frequency of stem-like PD-1^+^TCF1^+^CD8^+^ T cells. **i**, TDLN counts of stem-like PD-1^+^TCF1^+^CD8^+^ T cells. **j**, Counts of p15E tetramer^+^CD8^+^ TILs. **k**, Counts of p15E tetramer^+^CD8^+^ TILs expressing IFNγ. **l**, Counts of p15E tetramer^+^CD8^+^ TILs expressing CD25. **m**, Counts of p15E tetramer^+^CD8^+^ TILs expressing granzyme B. The mean ± s.d. values from stated replicates are shown. *P* values were determined by one-way ANOVA followed by Tukey’s multiple-comparison test. Exact *P* values are denoted in the figure. Source data

**Extended Data Fig. 10:**
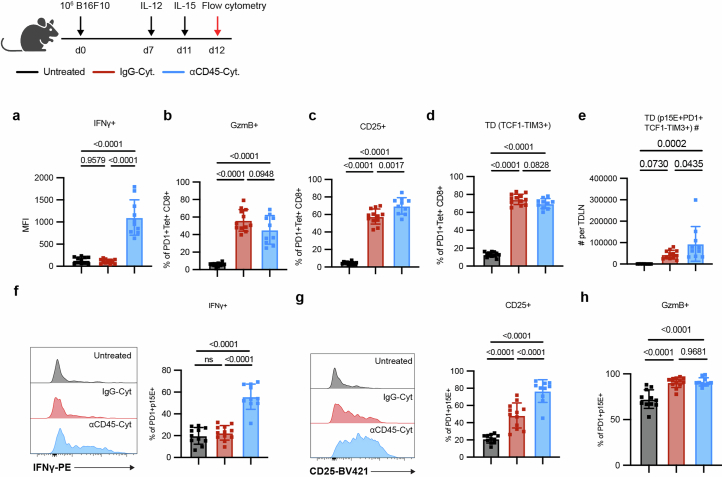
αCD45-Cyt therapy elicits potent effector phenotype on tumor-specific CD8^+^ T cells within the TDLN and TME. **a-h**. C57BL/6 J mice (n = 11 in untreated cohort, n = 12 in IgG-Cyt cohort, n = 10 in αCD45-Cyt cohort across two independent studies) inoculated with 10^6^ B16F10 tumor cells were treated with IgG-Cyt or αCD45-Cyt therapy on d7. Lymph nodes and tumors were harvested 24 hours after completion of therapy. **a-e** demonstrate the effects of αCD45-Cyt therapy on p15E^+^CD8^+^ T cells in the TDLN. Shown are **a**, IFNg MFI levels. **b**, Granzyme B expression. **c**, CD25 expression**. d**, Expression of terminally differentiated phenotype (TCF1^-^TIM3^+^) **e**, Absolute TDLN counts of terminally differentiated cells.**f-h** demonstrate the effects of αCD45-Cyt therapy on intratumoral p15E^+^CD8^+^ T cells. **f** Sample histogram of IFNg staining (left) and expression data (right). **g**, Sample histogram of CD25 staining (left) and expression data (right). **h** Granzyme B expression. Shown are mean ± s.d. P values were determined by one-way ANOVA followed by Tukey’s multiple comparison test. Exact P values are denoted in the figure; “ns” = not significant (P > 0.05). Source data

Recently, genetically induced sustained STAT5 activation has been shown to rewire exhausted T cells into a unique effector profile that enables control of chronic infections or tumors^27^. Based on our pathway analysis and in vitro data, we hypothesized that αCD45-IL-15 stimulation may therapeutically elicit similar sustained STAT5 induction and downstream generation of tumor-specific effectors. Probing the signaling dynamics of CD45 immunocytokines in the TDLNs 24 h after intratumoral dosing with αCD45-IL-15 alone revealed robust STAT5 phosphorylation induced in 80% of CD8^+^ T cells in the TDLN (Fig. 5a,b). By contrast, injection of IgG-IL-15 led to a dim pSTAT5^+^ population, closely mirroring the in vitro and RNA-seq results shown in Fig. 1e and 4d (Fig. 5a,b). Next, using an identical experimental set-up to that used in the RNA-seq analysis, we treated B16F10 tumors with αCD45-Cyt or IgG-Cyt therapy and profiled p15E^+^CD8^+^ T cells in the tumor and TDLNs 24 h after IL-15 dosing by flow cytometry. Only αCD45-Cyt treatment led to a statistically significant expansion of tetramer-positive CD8^+^ T cells in the TDLNs relative to untreated tumors (Fig. 5d,e). In addition to expanded numbers, TDLN tumor-specific cells primed by αCD45-Cyt exhibited a profound increase in IFNγ expression as measured by MFI and the number of IFN-γ^+^p15E^+^ cells (Fig. 5f,g and Extended Data Fig. 10a), whereas IgG-Cyt failed to elicit IFNγ production (Fig. 5f,g and Extended Data Fig. 10a). TDLN tumor-reactive cells also displayed high levels of granzyme B expression after treatment with either cytokine therapy (Extended Data Fig. 10b). Both treatments also significantly increased CD25 expression over untreated controls, but αCD45-Cyt treatment induced this upregulation on a larger fraction of the CD8^+^ compartment (Extended Data Fig. 10c).

Recent studies have suggested that antigen-specific TCF1^+^ stem-like T cells in disease-site-draining LNs play a critical role as a source of effector cell generation^25^^,^^27–32^. Given the robust effector response exhibited in treated TDLNs, we evaluated the impact of αCD45-Cyt therapy on the magnitude of the stem-like (T_SL_, TCF1^+^TIM3^−^) compartment. Although both treatments led to a drop in the relative proportion of TCF1^+^ T cells, the absolute number of tumor-specific, stem-like cells expressing PD-1^+^ and TCF1^+^ increased, with αCD45-Cyt therapy eliciting a sixfold higher count of stem-like cells in the TDLNs over untreated mice (Fig. 5h,i). Conversely, cytokine therapy skewed CD8^+^ T cells toward a terminally differentiated (TD, TCF1^−^TIM3^+^) phenotype as seen by high TIM3 expression in both treatment groups (Extended Data Fig. 10d). However, αCD45-Cyt therapy triggered a significantly larger expansion of the TD compartment by absolute count over IgG-Cyt therapy (Extended Data Fig. 10e).

In the treated tumor, αCD45-Cyt therapy led to increased numbers of tumor-reactive CD8^+^ T cells not seen after IgG-Cyt therapy (Fig. 5j). Treatment with αCD45-Cyt, but not IgG-Cyt, induced significantly higher levels of IFNγ within p15E-specific cells and significantly higher numbers of intratumoral IFNγ^+^CD8^+^cells (Extended Data Fig. 10f and Fig. 5k). Mice treated with αCD45-Cyt also displayed higher levels of CD25 expression, by both percentage and magnitude (Fig. 5l and Extended Data Fig. 10g). Intratumoral granzyme B expression was high across all groups, but only αCD45-Cyt therapy generated a significant increase in the compartment size (Fig. 5m and Extended Data Fig. 10h). Taken together, these immunophenotyping results show that local αCD45-Cyt therapy is not only able to dramatically alter the treated tumor immune microenvironment (TME), as other local therapies have accomplished, but also to reprogram the tumor-specific CD8^+^ population in the TDLNs, correlating with stronger abscopal responses.

## Discussion

Antibody-targeted cytokines have been pursued for many years with modest impact, resulting in part from the focus on targeting cancer cells lacking truly disease-specific antigens and the challenge that their biodistribution is often governed by the cytokine and not the antibody^5,33^. Recently, targeting immune cells through lineage or phenotypic markers has shown promising preclinical data^34,35^. In the present study, we focused on targeting host immune cells, using the unique biology of CD45 to develop antibody–cytokine fusions capable of modulating tumor-specific T cells within the TME and TDLNs. We leveraged CD45 as a unique cell-based ‘anchor’ for cytokines in two key ways: (1) its ability to be bound without triggering internalization; and (2) its abundant expression on leukocytes, with reported measurements upward of 100,000 molecules per cell^36^. We found evidence that these two characteristics allow for both *cis* and *trans* signaling of CD45-tethered cytokines. Thus, by decorating leukocytes in vivo with CD45-targeted immunocytokines, bystander cells lacking the cognate receptor can serve as cellular depots that ‘present’ the cytokine to nearby cells, extending the cytokine’s residence time. We show that specified intratumoral doses of CD45-targeted IL-15 and IL-12 are retained at the tumor and TDLNs for prolonged periods of time, leading to extended pSTAT5 signaling and acquisition of a potent effector program by tumor-reactive CD8^+^ T cells. This led to a reprogramming of the antitumor CD8^+^ T cell response at the TDLNs and robust systemic antitumor immunity. On a cytokine basis, the doses given in the αCD45-Cyt paradigm did not exceed 2 µg, a highly dose-sparing regimen highlighting the benefit of administration at the right time and place.

In striking contrast to the endogenous, unmanipulated, antitumor response, T cells within the αCD45-Cyt-treated TDLNs had transcriptional signatures resembling an acute antiviral response characterized by high expression of CD25, IFNγ, granzyme B and TIM3. These changes in T cell activation in the TDLNs also have many similarities with effects recently reported for antigen-specific T cells transduced to express constitutively active STAT5 (STAT5CA) in the setting of chronic LCMV infection^27^. In that case, splenic STAT5CA T cells were found to transition to a polyfunctional effector state, with depletion of the exhausted precursor TCF1^+^ subset and persist in the face of chronic infection. Prolonged signaling from cell-bound αCD45-IL-15 may represent a therapeutic approach to achieve similar effects in native T cells. Importantly, however, αCD45-Cyt therapy does not appear to deplete the tumor-specific, stem-like TCF1^+^PD-1^+^ subset that is thought to be the key self-renewing precursor of effector cells.

The ability to generate potent effectors at the TDLNs may explain the systemic response primed by αCD45-Cyt therapy. We hypothesize that tumor-reactive CD8^+^ T cells in TDLNs acted on by αCD45-Cyt therapy migrate into the systemic circulation and disseminate agnostically to distal lesions. We found evidence for a robust systemic response in two-tumor models of both MC38 colon carcinoma and B16F10 melanoma, as well as in two models of metastasis. These abscopal responses were not driven by drug leakage into the contralateral tumor, but were dependent on immune cell trafficking, because FTY720 ablated tumor control at the untreated lesion. These findings suggest that nodal cytokine retention after administration can prime potent antitumor immunity capable of systemic tumor control. A limitation of the present study is that we restricted our comparative studies to cytokines targeted to CD45 or administered as an irrelevant IgG fusion control. It will be important to determine how cytokine targeting to other receptors such as programmed cell death protein 1 (PD-1) or lineage markers such as CD8 compares with this delivery modality. However, for the specific case of localized immunotherapy, we expect the high copy number and unique internalization behavior of CD45 to provide distinct advantages for driving strong antitumor immunity.

αCD45-Cyt therapy is uniquely enabled by intratumoral administration as a conduit for LN exposure. Current interventional radiology and surgery techniques allow access to almost any surface or visceral lesion. Notably, the US Food and Administration Agency-approved oncolytic therapy talimogene laherparapvec (TVEC) is administered i.t. In addition, intranodal or peritumoral administration can be considered as an alternative administration route for TDLN exposure. Finally, while we explored some facets of CD45 biology, there are other potential aspects to consider. Differential splicing of human CD45 can correspond to distinct cell states, with shorter CD45 isoforms upregulated on CD8 T cell activation^37–39^. A CD45RO-specific antibody might therefore enable more targeted delivery of cytokines to activated T cells. In summary, our results highlight CD45 anchoring as a potent modular platform for enhancing the retention and response of immune agonists and provide rationale for further development of CD45 immunocytokines.

## Methods

### Cell lines

Cell lines CTLL-2 (American Type Culture Collection (ATCC)), Expi293F (Gibco) and HEK-Blue IL-12 (Invivogen) were cultured according to vendor instructions. MC38 (Kerafast, cat. no. ENH204-FP), MC38-ZsGreen (developed in the lab as described previously^40^) and B16F10 (ATCC, cat. no. CRL-6475) cells were cultured in Dulbecco’s modified Eagle’s medium supplemented with 10% fetal bovine serum (FBS), 100 units ml^−1^ of penicillin and 100 μg ml^−1^ of streptomycin. The 4T1-GFP-Luc (4T1-Luc) cells were generated by transduction of the 4T1 cell line (ATCC, cat. no. CRL-2539) with pGreenFire lentiviral vector (System Biosciences) as described previously^41^. The 4T1-Luc cells were cultured in Roswell Park Memorial Institute (RPMI)-1640 medium supplemented with 10% FBS, 100 units ml^−1^ of penicillin and 100 μg ml^−1^ of streptomycin. All cells were maintained at 37 °C and 5% CO_2_ and all were confirmed to be negative for *Mycoplasma* spp.

### Mice

All animal studies and procedures were carried out following federal, state and local guidelines under an institutional animal care and use committee-approved animal protocol by the Committee of Animal Care at MIT. For all studies involving animals, female BALB/c (Jackson Laboratory, cat. no. 000651), C57BL/6J (Jackson Laboratory, cat. no. 000664) and *Batf3*^*−/−*^ (Jackson Laboratory, cat. no. 013755) mice aged 6–8 weeks were purchased and maintained in the animal facility at Massachusetts Institute of Technology (MIT). Specific strains associated with experiments are listed in the figure captions. All mice were housed in a specific pathogen-free facility, and fed normal chow and water freely under standard animal facility conditions (12 h light:dark cycle). No statistical methods were used to predetermine sample sizes but our sample sizes are similar to those reported in previous publications^10–12,42,43^. Data collection and analysis were not performed blind to the conditions of the experiments.

### Cloning and protein purification

Gene blocks (gBlock, IDT) encoding for the light and heavy chain variable regions of anti-CD45 (clone 30-F11 (ref. ^44^)) or untargeted isotype control (anti-FITC, clone 4-4-20) were cloned into a mouse κ light chain and IgG2c backbone with LALA-PG^20^ mutations, respectively, in the mammalian expression vector gWiz (Genlantis). For αCD45-IL-15, murine IL-15/IL-15Rα_sushi_, as described previously^11^, was then cloned at the C terminus of the anti-CD45 (or anti-FITC control) heavy chain. To generate αCD45-IL-12, murine single chain IL-12 (scIL-12), as described previously^11^, was cloned at the amino terminus of the anti-CD45 (or anti-FITC control) heavy chain. For both IL-15 and IL-12 immunocytokines, a (Gly4Ser)_3_ linker was used between the cytokine and antibody. To generate human αCD45-IL-15, gBlocks encoding for the light and heavy chain variable regions of anti-human CD45 (clone BC8) were cloned into a human κ light chain and human IgG1 backbone with LALA-PG mutations, respectively. Human IL-15/IL-15Rα_sushi_, as described previously^11^, was then cloned at the C terminus of the anti-CD45 heavy chain.

A gBlock encoding for the extracellular domain of mouse CD45RO (used for ELISAs; sequence obtained from UniProt) was cloned into gWiz with a His6 tag. Plasmid sequences confirmed by Sanger sequencing (Quintara Biosciences) were transformed into Stellar Competent Cells (Takara Bio) and purified using the NucleoBond Xtra Midi endotoxin-free midi-prep kit (Takara Bio). For immunocytokines, an equal mass of heavy and light chain plasmids was transfected into Expi293F cells (Gibco) per the manufacturer’s instructions and harvested 6 d after transfection. All immunocytokines were purified using rProteinA Sepharose Fast Flow resin (Cytiva Life Sciences) and validated for size by sodium dodecylsulfate–polyacrylamide gel electrophoresis. His-tagged CD45RO was purified by TALON affinity resin (Takara) according to the manufacturer’s instructions. Purified proteins were confirmed to be endotoxin free (<0.1 endotoxin units (EU) per dose) using the Endosafe Nexgen-PTS system (Charles River). Purified proteins were flash frozen in liquid nitrogen and stored at −80 °C until use.

### ELISA and bioactivity assays

For ELISA assays, Maxisorp 96-well flat-bottomed plates (Thermo Fisher Scientific) were coated with recombinant CD45RO at 0.2 µg ml^−1^ in phosphate-buffered saline (PBS; Corning) overnight at 4 °C. Subsequent washes were performed with PBST (PBS supplemented with 0.05% v:v Tween-20 (Millipore-Sigma)). Blocking was performed in PBSTA (PBS supplemented with 1% w:v bovine serum albumin (BSA; Sigma-Aldrich) and 0.05% v:v Tween-20) overnight. Immunocytokines were diluted to relevant concentrations in PBSTA and detected via a horseradish peroxidase-conjugated anti-mouse IgG secondarily diluted 1:3,000 (Abcam). One-Step Ultra TMB-ELISA substrate solution (Thermo Fisher Scientific) was added to develop for 5 min and quenched with 2 N sulfuric acid (VWR). Absorbance at 450 nm (*A*_450_) with reference at 570 nm was measured on an Infinite M200 microplate reader (Tecan).

For IL-15 bioactivity, 10,000 CTLL-2 cells were seeded in a 96-well, U-bottomed plate in incomplete medium per the manufacturer’s instructions with stated IL-15 immunocytokine dilutions for 48 h at 37 °C. Proliferation was measured via CellTiter-Glo 2.0 Assay (Promega) following the manufacturer’s instructions. Luminescence was measured on a microplate reader (Tecan) with an integration time of 0.25 s. For the CD45 pre-blockade experiment, cells were incubated with 500 nM αCD45 (clone 30F11) for 4 h before the addition of IL-15 immunocytokine. IL-12 bioactivity was measured with HEK-Blue IL-12 reporter cells according to the manufacturer’s instructions (Invivogen).

### Primary CD8^+^ T cell preparation

Spleens from 6- to 8-week-old C57BL/6J female mice were harvested and processed into single-cell suspensions. CD8^+^ T cells were isolated using the EasySep Mouse CD8^+^ T cell isolation kit (StemCell Technologies) and resuspended at a concentration of 10^6^ cells ml^−1^ in complete RPMI supplemented with 1× sodium pyruvate (Thermo Fisher Scientific), 1× nonessential amino acids (Thermo Fisher Scientific) and 1× 2-mercaptoethanol (Thermo Fisher Scientific). Medium was additionally supplemented with 10 ng ml^−1^ of murine IL-2 (BioLegend) before resuspension and subsequent passaging. Isolated CD8^+^ T cells were activated for 48 h on a 6-well, nontissue culture-treated plate that was precoated with 0.5 µg ml^−1^ of anti-CD3 (BioXCell, clone 2C11) and 5 µg ml^−1^ of anti-CD28 (BioXCell, clone 37.51) overnight at 4 °C. The plate was washed twice before activation. After activation, T cells were cultured for 48 h before use in downstream experiments.

For human CD8^+^ T cell work, CD8^+^ T cells isolated from PBMCs were purchased from StemCell Technologies. Isolated cells were activated with αCD3/αCD28 Dynabeads (Thermo Fisher Scientific) at a 1:1 ratio in complete T cell medium (described above) supplemented with 10 ng ml^−1^ of human IL-2 for 72 h. Cells were then rested for at least 48 h before use in downstream assays.

### Fluorescence-quenching internalization assay

Internalization assays were performed and analyzed as described previously^45^. Briefly, immunocytokines were conjugated with Alexa Fluor-488 (AF488) using *N*-hydroxysuccinimide (NHS) ester chemistry (Invitrogen). Free dye was removed by Zeba spin desalting column purification (Thermo Fisher Scientific). Primary CD8 T cells, 100,000, were seeded in a 96-well plate and incubated with AF488-labeled immunocytokines at 10 µg ml^−1^ staggered at desired time points. Wells were then split such that one set was incubated with 25 µg ml^−1^ of anti-AF488-quenching antibody (Thermo Fisher Scientific, cat. no. A-11094) for 25 min. For human experiments, anti-human CD45 (clone MEM-28) conjugated to AF488 (Thermo Fisher Scientific) was used. IL15/IL15R was purchased from MedchemExpress. Viability was assessed via DAPI staining. The AF488 signal was measured using a BD LSR Fortessa and FACSDiva software and data were analyzed in FlowJo.

### Analysis of STAT phosphorylation by flow cytometry

For in vitro STAT5 experiments, primary CD8^+^ T cells cultured as described above were starved of IL-2 for 24 h and seeded into 96-well plates at 100,000 cells per well. IL-15 immunocytokines were added at 10 µg ml^−1^ for 1 h and subsequently washed with incomplete T cell medium (no IL-2) twice before resting for 24 h. IgG-IL-15 added at the same molar concentration (without washing) was used as a continuous control. Cells were immediately fixed in medium with equal volumes of BD Phosflow Fixation Buffer I prewarmed to 37 °C for 10 min. When required, cells were stained with Zombie Aqua viability stain for 5 min in PBS (1:1,000 dilution) before fixation. Cells were permeabilized for 30 min on ice with BD Phosflow Perm Buffer III that had been pre-chilled to −20 °C. Staining with anti-pSTAT5 antibodies (BD, clone 47) conjugated to AF647 or phycoerythrin (PE) was carried out at room temperature for 1 h. STAT4 experiments were performed identically, but used complete medium supplemented with IL-2 and immunocytokine incubation was performed at 2 µg ml^−1^ for 20 min. The pSTAT4 signal was detected with anti-pSTAT4 (BD, clone 38). For *trans*-signaling experiments, immunocytokines were conjugated with AF647 using NHS ester chemistry. Preloaded cells were labeled with CFSE per the manufacturer’s instructions. For *cis*-signaling experiments, CD8^+^ T cells were diluted with HEK293F (CD45^−^IL-15R^−^) at the stated ratios before incubation with αCD45-IL-15 at the stated concentrations. The total number of cells in the well was maintained at a constant level.

Measurement of STAT5 levels in vivo was carried out as previously described^46^. Briefly, TDLNs were processed into single-cell suspensions directly in BD Fixation Buffer I and samples were incubated at 37 °C for 10 min. Downstream permeabilization and staining were performed as described above. In all cases, the pSTAT signal was measured using a BD LSR Fortessa and FACSDiva software and data were analyzed in FlowJo.

### Tumor inoculation and treatment preparation

For all single-tumor experiments, mice aged 6–8 weeks were injected subcutaneously (s.c.) in the shaved right flank with 10^6^ tumor cells (MC38, MC38-ZsGreen or B16F10) in a volume of 50 μl of PBS. For two-tumor experiments, the contralateral tumor was inoculated on the left flank 3 d after the primary tumor, as stated in the study schematics. Before treatment, mice were randomized to ensure equal mean initial tumor size across groups. Immunocytokines were prepared at their stated doses (1 μg for IL-12 immunocytokines and 10 μg for IL-15 immunocytokines, where the mass indicated is the mass of the entire fusion protein) and dosed i.t. in 20 μl of PBS unless otherwise stated. Doses were informed by our biodistribution experiments as well as previous intratumoral cytokine work from our lab^10^. Peritumoral administration was performed in 50 μl of PBS injected s.c. at the tail base. Intraperitoneal administration was performed in 100 μl of PBS. The tumor area was calculated as the product of tumor length and width. For single-tumor studies, mice were euthanized when the tumor area exceeded 100 mm^2^; for two-tumor studies, mice were euthanized when cumulative tumor area exceeded 200 mm^2^. The maximal tumor burden was not exceeded on any study in this work. Immune memory rechallenge experiments were carried out 100 d after initial challenge with 10^5^ tumor cells on the left flank. Age-matched naive mice were used as controls for these studies. For the lung metastasis model used in Extended Data Fig. 8, 2 × 10^5^ B16F10 cells in 100 μl of PBS were inoculated retro-orbitally on the same day as the standard 10^6^ tumor-cell flank inoculation.

### 4T1 orthotopic inoculation and metastasis

For 4T1-Luc experiments, 0.5 × 10^6^ 4T1-Luc cells were inoculated into the fourth mammary fat pad of BALB/c mice. Then 12 d after the start of treatment, mice were anesthetized with isoflurane and provided preoperative, subcutaneous, sustained-release buprenorphine (1 mg kg^−1^; ZooPharm) and meloxicam (5 mg kg^−1^). The primary tumor was surgically removed and the wound was closed with surgical clips. Meloxicam (5 mg kg^−1^) was dosed every 24 h for 3 d postoperatively. Bioluminescence-imaging, 4T1-Luc tumor–bearing mice were monitored postoperatively for development of metastases using bioluminescence imaging beginning 1 week after surgical resection of the primary tumor. Animals were injected i.p. with 150 mg kg^−1^ of sterile filtered d-luciferin (PerkinElmer) in 200 ml of sterile PBS. Animals were imaged 15 min after the d-luciferin injection using the IVIS Spectrum Imagining System 100 (Xenogen).

### Tissue processing for flow cytometry

B16F10 or MC38 tumors were harvested, weighed and subsequently minced using dissection scissors in gentleMACS mouse tumor dissociation buffer (Miltenyi) prepared per the manufacturer’s instructions. As noted in the Miltenyi protocol, enzyme R was reduced to 20% of the stated amount to preserve surface epitope integrity. Minced tumors were processed on a gentleMACS Octo-dissociator with heaters (Miltenyi) using program mTDK_1 for B16F10 and mTDK_2 for MC38. Dissociated tumors were then filtered through a 70-μm strainer and 25 mg of tumor was plated for downstream staining. TDLNs were harvested, weighed and subsequently dissociated and filtered through a 5-ml round-bottomed tube with cell-strainer cap (Falcon) using the blunt rubber end of a 1-ml syringe plunger (Falcon). Then, 5 mg of TDLNs was used for downstream staining. Blood was collected by submandibular bleeding into MiniCollect K2-EDTA tubes (Greiner) and red blood cells were lysed using ACK Lysis Buffer (Gibco). When intracellular cytokine staining was performed, as in Fig. 5, samples were resuspended and plated in complete RPMI supplemented with 1× sodium pyruvate, 1× nonessential amino acids, 1× 2-mercaptoethanol and 1× brefeldin A (BioLegend) and allowed to incubate at 37 °C for 3 h before staining. Precision counting beads (50 μl, BioLegend) were added after initial resuspension and used for downstream data analysis. Viability was assessed with Zombie UV or Zombie NIR dyes (BioLegend, 1:1,000) in PBS for 20 min at room temperature. Subsequent washes and surface staining were performed in PBS supplemented with 1% BSA and 2 mM EDTA (Thermo Fisher Scientific). Samples were resuspended in Mouse Fc block Plus (BioLegend) before surface staining for 15 min. The antibodies against surface targets used are the following: CD3 (BD, cat. no. 612803, clone 17A3, 1:100), CD4 (BD, cat. no. 612923, clone GK1.5, 1:100), CD8 (BioLegend, cat. no. 100723, clone 53-6.7, 1:200), CD19 (BioLegend, cat. no. 115543, clone 6D5, 1:100), CD24 (BioLegend, cat. no. 101822, clone M1/69, 1:100), CD25 (BioLegend, cat. no. 102034, clone PC61, 1:100), CD44 (BD, cat. no. 612799, clone IM7, 1:100), CD45.2 (BD, clone: 104, 1:100), NK1.1 (BioLegend, cat. no. 108741, clone PK136, 1:100), MHC-II (BioLegend, cat. no. 107628, clone M5/114.15.2, 1:100), Ly6C (BioLegend, cat. no. 128036, clone HK1.4, 1:100), F4/80 (BioLegend, cat. no. 123147, clone BM8, 1:100), PD-1 (BioLegend, cat. no. 135220, clone 29F.1A12, 1:100) and TIM3 (BioLegend, cat. no. 119725, clone RMT3-23, 1:100). P15E tetramer (MBL) staining was performed in the presence of 50 nM dasatinib at a 1:75 dilution and anti-CD8 antibody clone KT15 (Thermo Fisher Scientific) was used to minimize background signal. Dasatinib incubation was not included in the staining mixture for the RNA-seq experiment. When performing intracellular staining, cells were fixed and permeabilized using the Foxp3 transcription buffer set (eBioscience). The samples against intracellular antigens used are as follows: TCF1 (Cell Signaling Technologies, cat. no. 90511, clone C63D9, 1:250), IFNγ (BioLegend, cat. no. 505808, clone XMG1.2, 1:200) and granzyme B (BioLegend, cat. no. 396418, clone QA16A02, 1:200). Intracellular staining was performed overnight at 4 °C. Cells were collected using a BD FACSymphony A3 and FACSDiva software and data were analyzed in FlowJo.

### Antibody-mediated cellular depletion

Immune cell depletions were carried out with antibodies targeting CD8a (BioXCell, clone 2.43, 400 μg twice weekly), NK1.1 (BioXCell, clone PK136, 400 μg twice weekly) and CSF1R (BioXCell, clone AFS98, 300 μg every other day) as previously described^11^. All depletions were given i.p. in 100 μl of PBS. Depletions were initiated 1 d before treatment and carried out for 4 weeks. Depletions were carried out in C57BL/6J mice unless otherwise noted.

### Tissue lysates for biodistribution and analyte measurement

IL-15 immunocytokines were labeled with AF647 using NHS ester chemistry per the manufacturer’s instructions. Free dye was removed with Zeba desalting columns. Molar amounts of dye for each immunocytokine were matched before dosing. Tissues were excised, weighed and processed in a GentleMACS M Tube using the program Protein_01 in radioimmunoprecipitation assay buffer (Thermo Fisher Scientific) supplemented with Halt Protease Inhibitor Mixture (Thermo Fisher Scientific). Samples were pelleted by centrifugation at 10,000*g* to remove debris and supernatants were pipetted on to a 384-well plate. Fluorescence was measured on a microplate reader (Tecan) with gain and *z*-value optimized for each tissue. Cytokine concentrations were calculated based on a standard curve prepared using known amounts of serially diluted AF647-labeled immunocytokine at the gain and *z*-value optimized for that given tissue. Serum mass density was taken to be 1 g ml^−1^ for conversion. The LEGENDplex mouse antivirus response panel (13-plex; BioLegend) was used following the vendor instructions.

### FTY720 preparation and dosing

FTY720 hydrochloride (Sigma-Aldrich) was stored in stock solutions at 10 mg ml^−1^ in dimethyl sulfoxide. Before treatment, stock solutions were diluted to a dose of 30 μg in 150 μl in PBS. In two-tumor MC38 studies, FTY720 was dosed every other day i.p. starting on day 5 after tumor inoculation.

### Leukocyte fraction analysis

To compare the CD45 infiltration of mouse and human tumors, we used existing datasets from The Cancer Genome Atlas (TIMER2.0 database^47^) or a comprehensive syngeneic mouse database (TISMO database^48^). The leukocyte fraction was calculated from EPIC RNA-seq analysis as the sum of B cell, NK cell, CD8, CD4 and macrophage fractions.

### Immunofluorescence staining

Inguinal LNs and tumors were harvested 24 h post-injection as described in Fig. 2a, embedded in optimal cutting temperature buffer (Fischer Scientific) and fresh frozen. Then 10-μm tissue sections were post-fixed with 4% paraformaldehyde (PFA) for 10 min, followed by three washes with PBS. Sections were incubated with Fc receptor blocker (Innovex) for 30 min and blocked for 1 h with 5% goat serum and 2.5% BSA in PBS. Staining with primary antibodies was performed overnight at 4 °C in blocking buffer (LN:IgD; BioLegend, cat. no. 405705) and CD8 (Abcam, cat. no. ab217344); tumors: CD8 and F4/80 (Abcam, cat. no. ab105156). After three washes with PBS, the sections were incubated with fluorochrome-conjugated secondary antibody (Thermo Fisher Scientific, cat. no. 35551) in blocking buffer for 30 min at room temperature. After three washes with PBS, the sections were mounted on to glass slides using mounting medium (ProLong Diamond Antifade Mountant, Thermo Fisher Scientific). High magnification images were acquired using a Leica SP8 laser-scanning confocal microscope equipped with a white light laser, a 405 solid state laser line and selective emission filters. Images were collected using a ×25 water immersion lens and a ×63 oil immersion lens.

### Immunohistochemistry staining of lung sections

Animals were euthanized and transcardially perfused with PBS before harvesting the lungs. Tissues were fixed overnight in 4% PFA at 4 °C, processed using conventional methods, embedded in paraffin and sectioned at 10 μm. Sections were then stained with hematoxylin and eosin and scanned using the Aperio Brightfield (Leica Biosystems) Slide Scanning System. The lung tissue and metastatic lesions were automatically detected via distinct pixel classifiers using QuPath v.0.4.3.

### CD8^+^ T cell RNA-seq, mapping and analysis

For RNA-seq experiments, 2,000–40,000 live CD3^+^CD8^+^CD44^+^p15E^+^ cells processed from TDLNs were sorted using a Sony MA900. RNA extraction was performed using the QIAGEN RNEasy Micro kit per the manufacturer’s instructions. RNA libraries were prepared using the Clontech SMARTer Stranded Total RNA-Seq Kit—Pico Input Mammalian and sequenced using the Illumina NextSeq500 75-nt kit. RNA-seq reads were aligned to the mouse genome with STAR (v.2.7.9a) using ensembl GRCm39 primary assembly as the reference. Aligned reads were quantified using RSEM (v.1.3.1) with ensembl GRCm39 (release 110) transcript annotations. The resulting counts were analyzed in R using DESeq2 for differential expression analysis, fgsea for GSEA and msigDB for the gene set database. Data visualization was done with ggplot2 and ComplexHeatmap. GSEA was utilized for the correlative analysis between our RNA-seq data and the gene expression signatures from Prokhnevska et al.^25^. The gene expression count matrix was obtained from National Center for Biotechnology Information’s Gene Expression Omnibus (GEO) with accession no. GSE216731. Differential gene expression analysis was performed on the LCMV Arm and TDLN groups and the genes were ranked by Wald’s test statistics. The ranked genes were compared with gene signatures from our data, specifically the upregulated genes in αCD45-Cyt versus untreated mice and the upregulated genes in untreated versus αCD45-Cyt mice. The enrichment score suggests the degree of correlation with T cells from either the LCMV Arm or TDLNs.

### Statistical methods

Statistics were computed in GraphPad Prism v.9 as denoted in the figure captions. For in vitro biodistribution and flow cytometry immunophenotyping experiments, comparisons were made by two-sided Student’s *t*-test or one- or two-way analysis of variance (ANOVA) followed by Tukey’s multiple-comparison test. Survival comparisons were made by log(rank) (Mantel–Cox) tests. Unless noted otherwise, data distributions were assumed to be normal but this was not formally tested. Differential gene expression analysis in the RNA-seq data was performed by two-sided Wald’s tests. In all RNA-seq analyses, *P* values are corrected by Benjamini–Hochberg to account for multiple hypothesis testing. No data/experiments were excluded unless there were technical issues with the experiment and outliers were not excluded. Exact *P* values are denoted in the figures. For all figures, NS is not significant (*P* > 0.05).

### Reporting summary

Further information on research design is available in the Nature Portfolio Reporting Summary linked to this article.

## Online content

Any methods, additional references, Nature Portfolio reporting summaries, source data, extended data, supplementary information, acknowledgements, peer review information; details of author contributions and competing interests; and statements of data and code availability are available at 10.1038/s41590-024-01925-7.

## Supplementary information

Supplementary Information Supplementary Fig. 1, and Tables 1 and 2.

Reporting Summary

## Source data

Source Data Figs. 1–5 Statistical source data.

Source Data Extended Data Figs. 1–10 Statistical source data.

Source Data Extended Data Figs. 1 and 3 Unprocessed gels.

## Data Availability

RNA-seq data were deposited into the GEO database under accession no. GSE252949. All other data supporting the findings of the present study are available within the paper and its Supplementary Information. Source data are provided with this paper.
