# Supplementary material for: Local delivery of cell surface-targeted immunocytokines programs systemic antitumor immunity
Source: Nat Immunol. 2024 Aug 7;25(10):1820–9. doi: 10.1038/s41590-024-01925-7 (PMC11436379; doi:10.1038/s41590-024-01925-7)
Supplement: Supplementary file 1 — Supplementary Fig. 1, and Tables 1 and 2. [file 41590_2024_1925_MOESM1_ESM.pdf]

nature immunology

Supplementary information

https://doi.org/10.1038/s41590-024-01925-7

Local delivery of cell surface-targeted immunocytokines programs systemic antitumor immunity

In the format provided by the authors and unedited

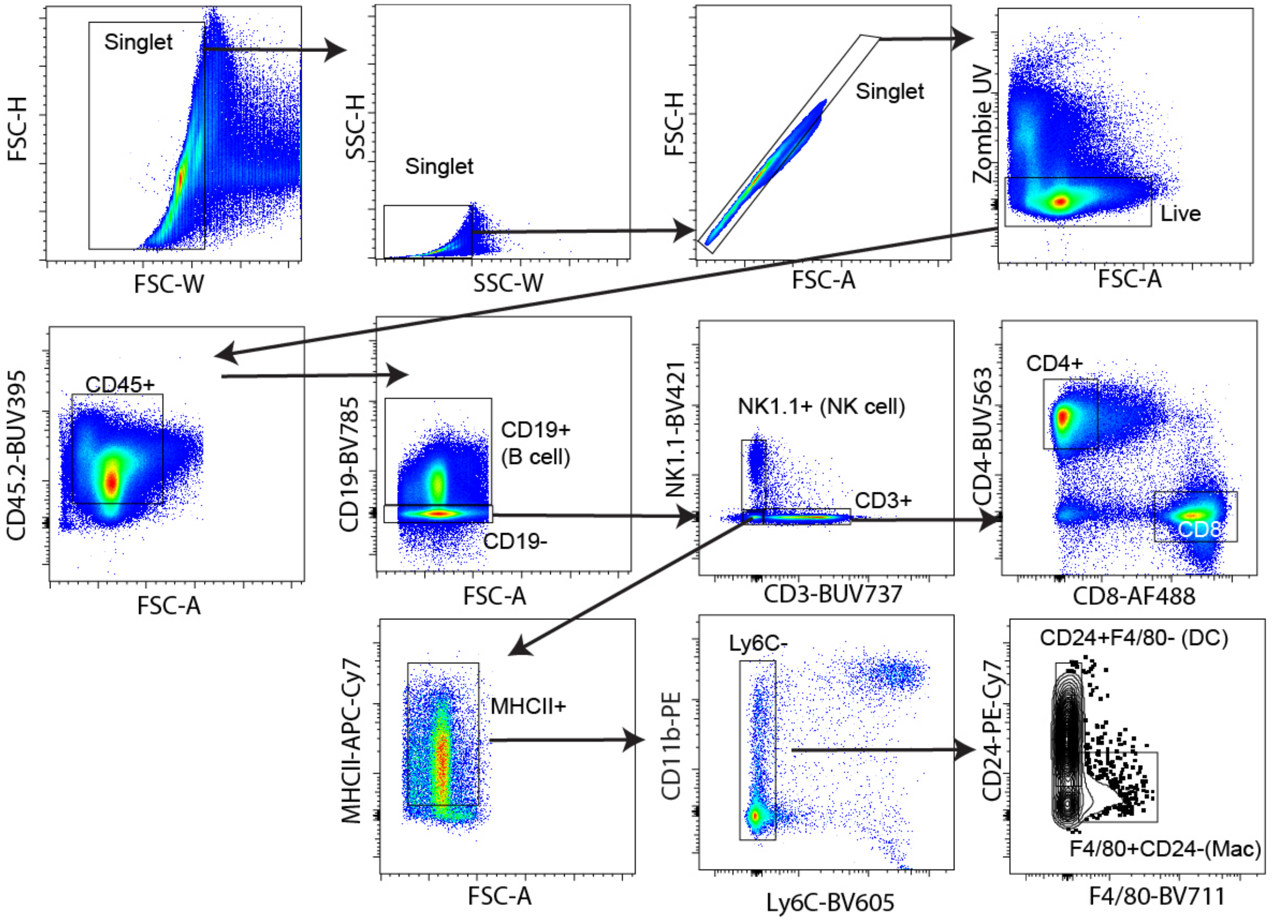

Supplementary Figure 1. Gating strategy used for lineage panel. Identical gating strategies were used for tumor and lymph node samples.

Supplementary Table 1. Amino acid sequences of mouse αCD45-cytokine fusions.
Key: V_H, V_L, linker, constant region, cytokine

αCD45-IL15 Light Chain (mCκ)	DIQLTQSPKSMSMSVGERVTLTCKASENVVTYVSWY<br>QQKPEQSPKLLIYGASNRYTGVPDRFTGSGSATDFTL<br>TISSVQAEDLADYHCGQGYSYPYTFGGGTKLEIK RAD<br>AAPTVSIFPPSSEQLTSGGASVVCFLNNFYPKDINVKW<br>KIDGSERQNGVLNSWTDQDSKDSTYSMSSTLTLTKDE<br>YERHNSYTCEATHKTSTSPIVKSFNRNEC
αCD45-IL15 Heavy Chain (mIgG2c LALA-PG)	QVQLLQSGGGLVQPGRSLKLSCLASGFIFSNYGMNWIR<br>QAPGKGLEWVASISSTSSYIQYADTVKGRFTISRENAKN<br>TLYLQMTSLISEDTALYYCARHGGYGYKGIWFAYWGQG<br>TLVTVSS AKTTAPSVYPLAPVCGGTTGSSVTLGCLVKGY<br>FPEPVTLTWNSGSLSSGVHTFPALLQSGLYTLSSSVTVT<br>SNTWPSQTITCNVAHPASSTKVDKKIEPRVPITQNPCPP<br>LKECPPCAAPDAAGGPSVFIFPPKIKDVLMISLSPMVTCV<br>VVDVSEDDPDVQISWFVNNVEVHTAQTQTHREDYNSTL<br>RVVSALPIQHQDWMSGKEFKCKVNNRALGSPIEKTISKP<br>RGPVRAPQVYVLPPPAEEMTKKEFSLTCMITGFLPAEIA<br>VDWTSNGRTEQNYKNTATVLDSDGSYFMYSKLRVQKS<br>TWERGSLFACSVVHEGLHNHLTTKTISRSLGK GGGGS<br>GTTCPPPVSIEHADIRVKNYSVNSRERYVCNSGFKRKAGT<br>STLIECVINKNTNVAHWTTPSLKCIRDPSLAGGSGGSGG<br>SGGSGGSGGSGGNWIDVRYDLEKIESLIQSIHIDTTLYTD<br>SDFHPSCKVTAMNCFLLELQVILHEYSNMTLNETVRNVL<br>YLANSTLSSNKNVAESGCKECEELEEKTFTEFLQSFIRIVQ<br>MFINTS
IgG-IL15 Light Chain (mCκ)	DVVMTQTPLSLPVSLGDQASISCRSSQSLVHSNG<br>NTYLRWYLQKPGQSPKVLIYKVSNRFSGVPDRFS<br>GSGSGTDFTLKISRVEAEDLGVYFCSQSTHVPWT<br>FGGGTKLEIK RADAAPTVSIFPPSSEQLTSGGASV<br>VCFLNNFYPKDINVKWKIDGSERQNGVLNSWTDQ<br>DSKDSTYSMSSTLTLTKDEYERHNSYTCEATHKTS<br>TSPIVKSFNRNEC
IgG-IL15 Heavy Chain (mIgG2c LALA-PG)	DVKLDETGGGLVQPGRPMKLSCVASGFTFSDYWMN<br>WVRQSPEKGLEWVAQIRNKPYNYETYYSDSVKGRFT<br>ISRDDSKSSVYLQMNNLRVEDMGIYYCTGSYYGMDY<br>WGQGTSVTVS AKTTAPSVYPLAPVCGGTTGSSVTLG<br>CLVKGYFPEPVTLTWNSGSLSSGVHTFPALLQSGLYT<br>LSSSVTVTSNTWPSQTITCNVAHPASSTKVDKKIEPRV<br>PITQNPCPPLKECPPCAAPDAAGGPSVFIFPPKIKDVL<br>MISLSPMVTCVVVDVSEDDPDVQISWFVNNVEVHTAQ<br>TQTHREDYNSTLRVVSALPIQHQDWMSGKEFKCKVNN<br>RALGSPIEKTISKPRGPVRAPQVYVLPPPAEEMTKKEFS<br>LTCMITGFLPAEIAVDWTSNGRTEQNYKNTATVLDSDG<br>SYFMYSKLRVQKSTWERGSLFACSVVHEGLHNHLTTK<br>TISRSLGK GGGGS GTTCPPPVSIEHADIRVKNYSVNSR<br>ERYVCNSGFKRKAGTSTLIECVINKNTNVAHWTTPSLK<br>CIRDPSLAGGSGGSGGSGGSGGSGGSGGNWIDVRYD<br>LEKIESLIQSIHIDTTLYTDSDFHPSCKVTAMNCFLLELQV<br>ILHEYSNMTLNETVRNVLYLANSTLSSNKNVAESGCKEC

	EELEEKTFTEFLQSFIRIVQMFINTS
αCD45-IL12 Light Chain (mCκ)	DIQLTQSPKSMSMSVGERVTLTCKASENVVTYVSWY QQKPEQSPKLLIYGASNRYTGVPDRFTGSGSATDFTL TISSVQAEDLADYHCGQGYSYPYTFGGGTKLEIK RAD AAPTVSIFPPSSEQLTSGGASVVCFLNNFYPKDINVKW KIDGSERQNGVLNSWTDQDSKDSTYSMSSTLTLTKDE YERHNSYTCEATHKTSTSPIVKSFNRNEC
αCD45-IL12 Heavy Chain (mIgG2c LALA-PG)	MWELEKDVYVVEVDWTPDAPGETVNLTCDTPEEDDIT WTSDQRHGVIGSGKTLTITVKEFLDAGQYTCHKGGET LSHSHLLLHKKENGIWSTEILKNFKNKTFLKCEAPNYS GRFTCSWLVQRNMDLKFNIKSSSSSPDSRAVTCGMAS LSAEKVTLDQRDYEKYSVSCQEDVTCPTAEETLPIELAL EARQQNKYENYSTSFFIRDIIKPDPPKNLQMKPLKNSQV EVSWEYPDSWSTPHSYFSLKFFVRIQRKKEKMKETEEG CNQKGAFLVEKTSTEVQCKGGNVCVQAQDRYYNSSCS KWACVPCRVRSGGSGGGSGGGSGGGSRVIPVSGPAR CLSQSRNLLKTTDDMVKTAREKLKHYSCTAEDIDHEDITR DQTSTLKTCLPLELHKNESCLATRETSSTTRGSCLPPQK TSLMMTLCLGSIYEDLKMYQTEFQAINAALQNHNHQQIILD KGMLVAIDELMQSLNHNGETLRQKPPVGEADPYRVKMKL CILLHAFSTRVVTINRVMGYLSSA GGGGSGGGGSGGGGS QVQLLQSGGGLVQPGRSLKLSCLASGFIFSNYGMNWIRQ APGKGLEWVASISSTSSYIQYADTVKGRFTISRENAKNTLY LQMTSLISEDTALYYCARHGGYGYKGIWFAYWGQGTLV TVSS AKTTAPSVYPLAPVCGGTTGSSVTLGCLVKGYFPE PVTLTWNSGSLSSGVHTFPALLQSGLYTLSSSVTVTSNT WPSQTITCNVAHPASSTKVDKKIEPRVPITQNPCPPLKEC PPCAAPDAAGGPSVFIFPPKIKDVLMISLSPMVTCVVVDVS EDDPDVQISWFVNNVEVHTAQTQTHREDYNSTLRVVSALP IQHQDWMSGKEFKCKVNNRALGSPIEKTISKPRGPVRAP QVYVLPPPAEEMTKKEFSLTCMITGFLPAEIAVDWTSNGRT EQNYKNTATVLDSDGSYFMYSKLRVQKSTWERGSLFACSV VHEGLHNHLTTKTISRSLGK
IgG-IL12 Light Chain (mCκ)	DVVMTQTPLSLPVSLGDQASISCRSSQSLVHSNG NTYLRWYLQKPGQSPKVLIYKVSNRFSGVPDRFS GSGSGTDFTLKISRVEAEDLGVYFCSQSTHVPWT FGGGTKLEIK RADAAPTVSIFPPSSEQLTSGGASV VCFLNNFYPKDINVKWKIDGSERQNGVLNSWTDQ DSKDSTYSMSSTLTLTKDEYERHNSYTCEATHKTS TSPIVKSFNRNEC
IgG-IL12 Heavy Chain (mIgG2c LALA-PG)	MWELEKDVYVVEVDWTPDAPGETVNLTCDTPEED DITWTSDQRHGVIGSGKTLTITVKEFLDAGQYTCHK GGETLSHSHLLLHKKENGIWSTEILKNFKNKTFLKC EAPNYSGRFTCSWLVQRNMDLKFNIKSSSSSPDSR AVTCGMASLSAEKVTLDQRDYEKYSVSCQEDVTCP TAEETLPIELALEARQQNKYENYSTSFFIRDIIKPDPP KNLQMKPLKNSQVEVSWEYPDSWSTPHSYFSLKFF VRIQRKKEKMKETEEGCNQKGAFLVEKTSTEVQCK GGNVCVQAQDRYYNSSCSKWACVPCRVRSGGSG GGSGGGSGGGSRVIPVSGPARCLSQSRNLLKTTDD

	MVKTAREKLKHYSCTAEDIDHEDITRDQTSTLKTCLP LELHKNESCLATRETSSTTRGSCLPPQKTSLMMTLC LGSIYEDLKMYQTEFQAINAALQNHNHQQIILDKGML VAIDELMQSLNHNGETLRQKPPVGEADPYRVKMKL CILLHAFSTRVVTINRVMGYLSSA GGGGSGGGGSG GGGS DVKLDETGGGLVQPGRPMKLSCVASGFTFS DYWMNWVRQSPEKGLEWVAQIRNKPYNYETYYSD SVKGRFTISRDDSKSSVYLQMNNLRVEDMGIYYCTG SYYGMDYWGQGTSVTVS AKTTAPSVYPLAPVCGGT TGSSVTLGCLVKGYFPEPVTLTWNSGSLSSGVHTFP ALLQSGLYTLSSSVTVTSNTWPSQTITCNVAHPASST KVDKKIEPRVPITQNPCPPLKECPPCAAPDAAGGPSV FIFPPKIKDVLMISLSPMVTCVVVDVSEDDPDVQISWF VNNVEVHTAQTQTHREDYNSTLRVVSALPIQHQDWM SGKEFKCKVNNRALGSPIEKTISKPRGPVRAPQVYVLP PPAEEMTKKEFSLTCMITGFLPAEIAVDWTSNGRTEQN YKNTATVLDSDGSYFMYSKLRVQKSTWERGSLFACSV VHEGLHNHLTTKTISRSLGK

Supplementary Table 2. Amino acid sequences of human αCD45-IL15 fusions

Key: V_H/V_L, linker, constant region, cytokine

Human αCD45-IL15 Light Chain (hCκ)	DIALTQSPASLAVSLGQRATISCRASKSVSTSGYSYLHW YQQKPGQPPKLLIYLASNLESGVPARFSGSGSGTDFTLN IHPVEEEDAATYYCQHSRELPFTFGSGTKLEIK RTVAAPS VFIFPPSDEQLKSGTASVVCLLNNFYPREAKVQWKVDNA LQSGNSQESVTEQDSKDSTYSLSSTLTLSKADYEKHKVY ACEVTHQGLSSPVTKSFNRGEC
Human αCD45-IL15 Heavy Chain (hIgG1 LALA-PG)	EVKLLESGGGLVQPGGSLKLSCAASGFDFSRYWMSWVR QAPGKGLEWIGEINPTSSTINFTPSLKDKVFISRDNAKNTL YLQMSKVRSEDTALYYCARGNYYRYGDAMDYWGQGTSV TVSS ASTKGPSVFPLAPSSKSTSGGTAALGCLVKDYFPEP VTVSWNSGALTSGVHTFPAVLQSSGLYSLSSVVTVPSSSL GTQTYICNVNHKPSNTKVDKKVEPKSCDKTHTCPPCPAPE AAGGPSVFLFPPKPKDTLMISRTPEVTCVVVDVSHEDPEV KFNWYVDGVEVHNAKTKPREEQYNSTYRVVSVLTVLHQD WLNGKEYKCKVSNKALGAPIEKTISKAKGQPREPQVYTLPP SRDELTKNQVSLTCLVKGFYPSDIAVEWESNGQPENNYKT TPPVLDSDGSFFLYSKLTVDKSRWQQGNVFSCSVMHEALH NHYTQKSLSLSPGK GGGS ITCPPPMSVEHADIWVKSYSLYS RERYICNSGFKRKAGTSSLTECVLNKATNVAHWTTPSLKCIR GGSGGSGGSGGSGGSGGSGGNWVNVISDLKKIEDLIQSMHI DATLYTESDVHPSCKVTAMKCFLLELQVISLESGDASIHDTVE NLIILANDSLSSNGNVTESGCKECEELEEKNIKEFLQSFVHIVQ MFINTSHHHHHH
