# Supplementary material for: Local delivery of cell surface-targeted immunocytokines programs systemic antitumor immunity
Source: Nat Immunol. 2024 Aug 7;25(10):1820–9. doi: 10.1038/s41590-024-01925-7 (PMC11436379; doi:10.1038/s41590-024-01925-7)
Supplement: Supplementary file 2 — Reporting Summary [file 41590_2024_1925_MOESM2_ESM.pdf]

nature portfolio

Corresponding author(s): Darrell J. Irvine, K. Dane Wittrup

Last updated by author(s): Jul 6, 2024

Reporting Summary

Nature Portfolio wishes to improve the reproducibility of the work that we publish. This form provides structure for consistency and transparency in reporting. For further information on Nature Portfolio policies, see our Editorial Policies and the Editorial Policy Checklist.

Statistics

For all statistical analyses, confirm that the following items are present in the figure legend, table legend, main text, or Methods section.

n/a	Confirmed	
☐	☒	The exact sample size (*n*) for each experimental group/condition, given as a discrete number and unit of measurement
☐	☒	A statement on whether measurements were taken from distinct samples or whether the same sample was measured repeatedly
☐	☒	The statistical test(s) used AND whether they are one- or two-sided<br>*Only common tests should be described solely by name; describe more complex techniques in the Methods section.*
☒	☐	A description of all covariates tested
☐	☒	A description of any assumptions or corrections, such as tests of normality and adjustment for multiple comparisons
☐	☒	A full description of the statistical parameters including central tendency (e.g. means) or other basic estimates (e.g. regression coefficient) AND variation (e.g. standard deviation) or associated estimates of uncertainty (e.g. confidence intervals)
☐	☒	For null hypothesis testing, the test statistic (e.g. *F*, *t*, *r*) with confidence intervals, effect sizes, degrees of freedom and *P* value noted<br>*Give P values as exact values whenever suitable.*
☒	☐	For Bayesian analysis, information on the choice of priors and Markov chain Monte Carlo settings
☒	☐	For hierarchical and complex designs, identification of the appropriate level for tests and full reporting of outcomes
☒	☐	Estimates of effect sizes (e.g. Cohen's *d*, Pearson's *r*), indicating how they were calculated

Our web collection on statistics for biologists contains articles on many of the points above.

Software and code

Policy information about availability of computer code

Data collection	Flow cytometry data were obtained using BD FACSDiva software (v7). FACS data were obtained using a Sony MA900 and accompanying software. Plate-based fluorescent/absorbance data were measured using the Tecan Infinite M200 Pro plate reader and accompanying software. Confocal images were taken on a Leica SP8 laser scanning confocal microscope and associated LasX software (v1.4.6). RNAseq data were obtained using an Illumina NextSeq500.
Data analysis	FlowJo 10.8.2 was used for the analysis of flow cytometry data. GraphPad Prism 10.1 was used for plotting and statistical analysis. RNA-seq analysis was performed in R(v4.3.2) using DESeq2 v1.42.1, fgsea v1.29.1, msigdbr v7.5.1, ggplot2 v3.5.0 for individual analysis. Image analysis for microscopy was performed using Qupath v0.4.3. Microsoft Excel, Microsoft Word (v16.8), and Adobe Illustrator (v27.7) were used to draft the manuscript.

For manuscripts utilizing custom algorithms or software that are central to the research but not yet described in published literature, software must be made available to editors and reviewers. We strongly encourage code deposition in a community repository (e.g. GitHub). See the Nature Portfolio guidelines for submitting code & software for further information.

Data

Policy information about availability of data

All manuscripts must include a data availability statement. This statement should provide the following information, where applicable:

- Accession codes, unique identifiers, or web links for publicly available datasets
- A description of any restrictions on data availability
- For clinical datasets or third party data, please ensure that the statement adheres to our policy

RNA-seq data were deposited into the Gene Expression Omnibus database under accession number GSE252949. RNAseq reads were aligned to the mouse genome with STAR (v2.7.9a) using ensembl GRCm39 primary assembly as the reference (https://useast.ensembl.org/Mus_musculus/Info/Index). Data supporting the findings of this study are available as source data are provided with this paper.

Research involving human participants, their data, or biological material

Policy information about studies with human participants or human data. See also policy information about sex, gender (identity/presentation), and sexual orientation and race, ethnicity and racism.

Reporting on sex and gender	N/A
Reporting on race, ethnicity, or other socially relevant groupings	N/A
Population characteristics	N/A
Recruitment	N/A
Ethics oversight	N/A

Note that full information on the approval of the study protocol must also be provided in the manuscript.

Field-specific reporting

Please select the one below that is the best fit for your research. If you are not sure, read the appropriate sections before making your selection.

☒ Life sciences ☐ Behavioural & social sciences ☐ Ecological, evolutionary & environmental sciences

For a reference copy of the document with all sections, see nature.com/documents/nr-reporting-summary-flat.pdf

Life sciences study design

All studies must disclose on these points even when the disclosure is negative.

Sample size	No statistical methods were used to pre-determine sample sizes but our sample sizes are similar to those reported in previous publications (Moynihan et al. Nat Med 22, 1402–1410 (2016); Agarwal et al. Nat Biomed Eng 6, 129–143 (2022); Zhang et al. Nat. Biomed. Eng 7, 1113–1128 (2023); Momin et al, Science Translational Medicine 11.498 (2019): eaaw2614.; Wang et al. Cell reports 37.8 (2021)).
Data exclusions	No data were excluded.
Replication	Experiments were repeated to confirm reproducibility, with specific sample sizes and details indicated in figure captions.
Randomization	For all tumor studies, animals were randomized to ensure the average tumor burden was equal across treatment groups.
Blinding	Investigators were not blinded to treatment for logistical reasons.

Reporting for specific materials, systems and methods

We require information from authors about some types of materials, experimental systems and methods used in many studies. Here, indicate whether each material, system or method listed is relevant to your study. If you are not sure if a list item applies to your research, read the appropriate section before selecting a response.

Materials & experimental systems

n/a	Involved in the study
☐	☒ Antibodies
☐	☒ Eukaryotic cell lines
☒	☐ Palaeontology and archaeology
☐	☒ Animals and other organisms
☒	☐ Clinical data
☒	☐ Dual use research of concern
☒	☐ Plants

Methods

n/a	Involved in the study
☒	☐ ChIP-seq
☐	☒ Flow cytometry
☒	☐ MRI-based neuroimaging

Antibodies

Antibodies used

In vitro T cell activation: anti-mCD3 (BioXCell, Cat. #BE0001-1, Clone 145-2C11); anti-mCD28 (BioXCell, Cat. #BE0015-1, Clone 37.51). Antibodies were used at concentrations as described in the Methods.

Fluorescence-quenching assay: anti-AF488 quenching antibody (ThermoFisher, Cat. A-11094)

ELISA: HRP-conjugated anti-mouse IgG secondary diluted 1:3000 (Abcam, Cat. ab97023)

Phospho-STAT staining: anti-pSTAT5 (clone 47, BD, Cat 612599), anti-pSTAT4 (clone 38, BD, Cat 558137)

Flow cytometry (all mouse targets): CD3-BUV737 (17A2, BD 612803, 1:100), CD4-BUV563 (GK1.5, BD 612923, 1:100), CD8-AF488 (53-6.7, BioLegend 100723 1:200), CD19-BV785 (6D5, BioLegend 115543, 1:100), CD24-PE-Cy7 (M1/69, BioLegend 101822, 1:100), CD25-BV421 (PC61, BioLegend 102034, 1:100), CD44-BUV737 (IM7, BD 612799, 1:100), CD45.2-BUV395 (BD 564616, clone: 104, 1:100), NK1.1-BV421 (PK136, BioLegend 108741, 1:100), MHCII-APC-Cy7 (M5/114.15.2, BioLegend 107628, 1:100), Ly6C-BV605 (HK1.4, BioLegend 128036, 1:100), F4/80-BV711 (BM8, BioLegend 123147, 1:100), PD1-BV605 (29F.1A12, BioLegend 135220, 1:100), TIM3-BV785 (RMT3-23, BioLegend 119725, 1:100). P15E-APC tetramer (MBL TB-M507-2) staining was performed in the presence of 50 nM dasatinib at a 1:75 dilution and anti-CD8 antibody clone KT15 (ThermoFisher) was used to minimize background signal. Dasatinib incubation was not included in the staining mixture for the RNAseq experiment. When performing intracellular staining, cells were fixed and permeabilized using the Foxp3 transcription buffer set (eBioscience). Samples against intracellular antigens used as following: TCF1-PE-Cy7 (C63D9, Cell Signaling Technologies 90511, 1:250), IFNg-PE (XMG1.2, BioLegend 505808, 1:200), Granzyme B-APC-Fire750 (QA16A02, BioLegend 396418, 1:200). Intracellular staining was performed overnight at 4C.

Antibody depletions: Immune cell depletions were carried out with antibodies targeting CD8a (BioXcell BE0061, Clone 2.43, 400 µg twice weekly), NK1.1 (BioXCell BE0036, clone PK136, 400 µg twice weekly), CSF1R (BioXCell BE0213, clone AFS98, 300 µg every other day)

Histology: IgD (Biolegend, clone 11-26c.2a, 405705, 1:50), CD8 (Abcam, clone EPR21769, ab217344, 1:50), F4/80 (Abcam, clone CI:A3-1, ab105156, 1:50).

Validation

All antibodies used were commercially available and confirmed to have validation statements from the manufacturer for their relevant use in this study.

Eukaryotic cell lines

Policy information about cell lines and Sex and Gender in Research

Cell line source(s)

CTLL-2, B16F10 cells, and 4T1 cells were purchased from ATCC. HEK293F and Expi293 cells were purchased from Gibco. HEK-Blue IL-12 cells were purchased from Invivogen. MC38 cells were purchased from Kerafast. MC38-ZsGreen were generated in-house as described in a previous publication (Dane et al., Nature Materials 21.6 (2022): 710-720). 4T1-GFP-Luc (4T1-Luc) cells were generated by transduction of the 4T1 cell line (ATCC, CRL-2539) as described previously (Milling et al., Cancer Immunol. Res. 10, 26-39 (2022)).

Authentication

Each cell line was maintained separately and stocked in early passages to minimize contamination and to preserve cell identity. No further in-house authentication was performed after procuring cell lines.

Mycoplasma contamination

The cell lines were periodically tested and confirmed to be free of mycoplasma contamination.

Commonly misidentified lines
(See ICLAC register)

No commonly misidentified cell lines were used.

Animals and other research organisms

Policy information about studies involving animals; ARRIVE guidelines recommended for reporting animal research, and Sex and Gender in Research

Laboratory animals

Female C57BL/6, Batf3–/– , and BALB/c mice were all obtained from Jackson Laboratories. Mice were between 6 and 8 weeks old at

Laboratory animals

the start of all studies and weighed approximately 18-20 g. Mice were housed in a specific-pathogen free facility and fed normal chow and water ad libitum under standard animal facility conditions (12 hour light/dark cycle, temperature of 22 C, relative humidity of 40%-70%.

Wild animals

The study did not involve wild animals.

Reporting on sex

All studies using mice employed female mice.

Field-collected samples

The study did not involve samples collected from the field.

Ethics oversight

All animal studies and procedures were carried out following federal, state and local guidelines under an animal protocol approved by the institutional animal care and use committee at MIT.

Note that full information on the approval of the study protocol must also be provided in the manuscript.

Plants

Seed stocks

Report on the source of all seed stocks or other plant material used. If applicable, state the seed stock centre and catalogue number. If plant specimens were collected from the field, describe the collection location, date and sampling procedures.

Novel plant genotypes

Describe the methods by which all novel plant genotypes were produced. This includes those generated by transgenic approaches, gene editing, chemical/radiation-based mutagenesis and hybridization. For transgenic lines, describe the transformation method, the number of independent lines analyzed and the generation upon which experiments were performed. For gene-edited lines, describe the editor used, the endogenous sequence targeted for editing, the targeting guide RNA sequence (if applicable) and how the editor was applied.

Authentication

Describe any authentication procedures for each seed stock used or novel genotype generated. Describe any experiments used to assess the effect of a mutation and, where applicable, how potential secondary effects (e.g. second site T-DNA insertions, mosiacism, off-target gene editing) were examined.

Flow Cytometry

Plots

Confirm that:

☒ The axis labels state the marker and fluorochrome used (e.g. CD4-FITC).

☒ The axis scales are clearly visible. Include numbers along axes only for bottom left plot of group (a 'group' is an analysis of identical markers).

☒ All plots are contour plots with outliers or pseudocolor plots.

☒ A numerical value for number of cells or percentage (with statistics) is provided.

Methodology

Sample preparation

B16F10 or MC38 tumors were harvested, weighed, and subsequently minced using dissection scissors in gentleMACS mouse tumor dissociation buffer (Miltenyi) prepared per manufacturer's instructions. As noted in the Miltenyi protocol, Enzyme R was reduced to 20% of the stated amount to preserve surface epitope integrity. Minced tumors were processed on a gentleMACS Octo-dissociator with heaters (Miltenyi) using program mTDK_1 for B16F10 and mTDK_2 for MC38. Dissociated tumors were then filtered through a 70-micron strainer and 25 mg tumor was plated for downstream staining. TDLN were harvested, weighed, and subsequently dissociated and filtered through a 5 mL round-bottom tube with cell-strainer cap (Falcon) using the blunt rubber end of a 1mL syringe plunger (Falcon). 5 mg of TDLN was used for downstream staining. Blood was collected by sub-mandibular bleeding into MiniCollect K2-EDTA tubes (Greiner) and red blood cells were lysed using ACK Lysis Buffer (Gibco). When intracellular cytokine staining (ICS) was performed, as in Fig. 5, samples were resuspended and plated in complete RPMI supplemented with 1X sodium pyruvate (ThermoFisher), 1X non-essential amino acids (ThermoFisher), 1X beta-mercaptoethanol (ThermoFisher), and 1X brefeldin A (BioLegend) and allowed to incubate at 37C for 3 hours prior to staining. Precision counting beads (50uL, BioLegend) were added after initial resuspension and used for downstream data analysis.

Instrument

Cells were analysed using BD FACS LSR Fortessa or BD FACS Symphony A3 flow cytometers.

Software

BD FACSDiva was used for the collection of FACS data and FlowJo was used for data analysis. The collected data were plotted with statistical analysis by GraphPad Prism.

Cell population abundance

The purity of the sorted cells was more than 95%

Gating strategy

Briefly, cells were gated on FSC-A vs SSC-A to exclude debris and then FSC-H vs FSC-W followed by SSC-H vs SSC-W to gate on single cells and exclude doublets. Cells were than gated on viability vs FSC-A to exclude dead cells. For lineage gating, immune cells were gated as CD45+. Immune cells were then gated on CD19 vs FSC-A to discern B cells from other cells. CD19 negative cells were gated on NK1.1 vs CD3 to identify NK cells and T cells. T cells were then subgated by CD4 vs CD8. CD3-CD19- cells were then gated on MHCII+, Ly6C-,CD24+F4/80- as dendritic cells or CD24-F4/80+ as macrophages. This is summarized in Supplementary Information. Phenotypic gating is shown in each relevant figure.

☒ Tick this box to confirm that a figure exemplifying the gating strategy is provided in the Supplementary Information.
