# Supplementary figures and images for: Local delivery of cell surface-targeted immunocytokines programs systemic antitumor immunity
Source: Nat Immunol. 2024 Aug 7;25(10):1820–9. doi: 10.1038/s41590-024-01925-7 (PMC11436379; doi:10.1038/s41590-024-01925-7)

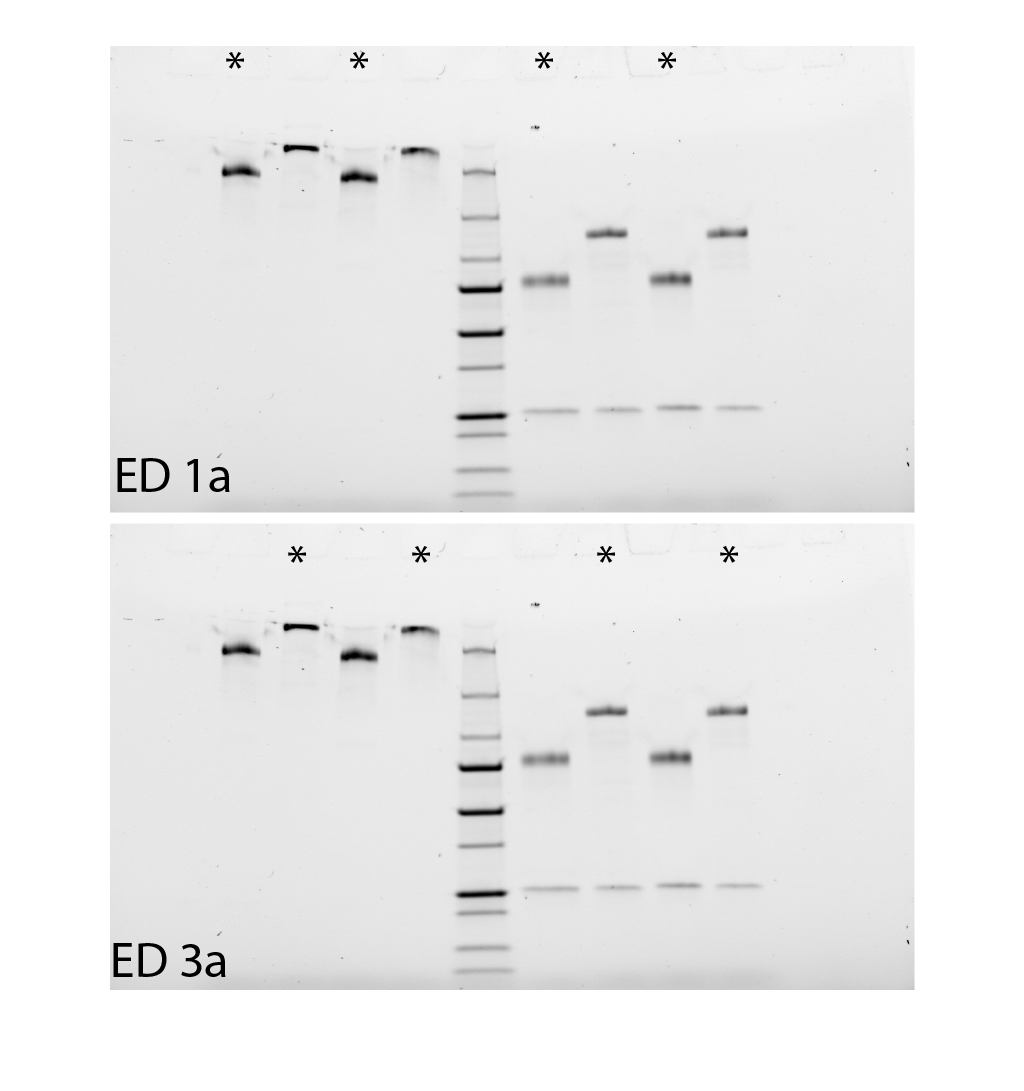

Supplement: Supplementary file 5 — Unprocessed gels. [file 41590_2024_1925_MOESM5_ESM.jpg]
